# Identification and pathogenicity of six fungal species causing canker and dieback disease on golden rain tree in Beijing, China

**DOI:** 10.1080/21501203.2022.2096144

**Published:** 2022-07-05

**Authors:** Meng Pan, Lu Lin, Chengming Tian, Xinlei Fan

**Affiliations:** The Key Laboratory for Silviculture and Conservation of Ministry of Education, Beijing Forestry University, Beijing, China

**Keywords:** Ascomycota, morphology, plant pathogen, phylogeny, taxonomy

## Abstract

Golden rain trees (*Koelreuteria paniculata*) are largely cultivated because of their important ornamental, medicinal, and economic value. However, they are affected by canker and dieback disease to a large extent. To determine the fungi associated with canker and dieback disease of golden rain trees, isolations were obtained from diseased branches and twigs during 2019 and 2020 in greenbelts and nurseries in Beijing, China. Isolates were identified as six species (*Allocryptovalsa castaneicola, Botryosphaeria dothidea, Cytospora koelreutericola* sp. nov., *Dothiorella acericola, Eutypella citricola*, and *Peroneutypa scoparia*) based on morphological features and phylogenetic analyses of ITS, *act, rpb2, tef1-α*, and *tub2*. The results of pathogenicity tests indicated that all fungi produced discoloration and *Botryosphaeria dothidea* was highly aggressive to golden rain tree. In conclusion, this study explored the taxonomy, phylogeny, and pathogenicity of different fungal species associated with canker and dieback disease on golden rain tree and provided fundamental knowledge to improve disease management.

## Introduction

Golden rain tree (*Koelreuteria paniculata*) known as lantern tree in China is a deciduous tree species in Sapindaceae, which is one of the most ideal and important tree species for landscape gardening (Qiu et al. 2014; Tang 2015). This tree species also has a high economic value. Its trunk can be used to extract tannin, its flowers can be used to make dyes, and its seeds can be used to squeeze oil (Li et al. 2020). The substances secreted by its leaves have inhibitory effects on a variety of fungi and bacteria, which antibacterial effect is not lower than antibacterial and anti-inflammatory drugs (Ma et al. 1998; Yang et al. 2000; Liu et al. 2006). In addition, golden rain trees can effectively reduce the concentration of heavy metals and play an important role in improving scenery (Tian et al. 2009). So far, golden rain trees are cultivated all over the world because of the quite tolerant of wide range of soil conditions, drought, heat, wind, and air pollution (Rehman and Park 2000; Kamala-Kannan et al. 2010).

Although golden rain trees have been planted as an ornamental tree in temperate regions across the world, the survival is threatened by various pathogens. *Juxtiphoma eupyrena* has been reported to cause leaf spot disease on *K. paniculata* (Chandel and Kumar 2017). In Italy, the diseased golden rain trees infected with *Verticillium* species showed yellowing of the leaves and twig dieback (Polizzi et al. 2007). Recent studies indicated that *Bulbouncinula, Erysiphe, Typhulochaeta*, and *Uncinula* were shown to cause powdery mildew on *K. paniculata* (Tai 1979; Zheng and Yu 1987; Chen 2003; Liu et al. 2020). Apart from the above, canker and dieback diseases of golden rain tree are commonly found in nature but have rarely been reported (Jaklitsch and Voglmayr 2014; Baysal-Gurel et al. 2020).

Canker and dieback diseases of woody hosts are common, which can cause the host to fail to grow normally, reduce the yield and longevity of orchards and nurseries, and eventually lead to replanting in the later stage (Abe et al. 2007; Gramaje et al. 2012; Pan et al. 2021a). In recent years, *Lasiodiplodia theobromae* (Wang et al. 2012) and *Neofusicoccum parvum* (Li et al. 2020) were isolated from blighted branches and stems of golden rain trees in China, respectively. Baysal-Gurel et al. (2020) also indicated that *L. theobromae* caused stem canker of golden rain trees in commercial nursery in the United States. *Thyronectria rhodochlora* was reported from dead corticated twigs on the ground or attached to the trees in Australia (Jaklitsch and Voglmayr 2014). In order to accomplish better disease control, it is necessary to explore the diversity and pathogenicity of potential pathogens under the current situation that a lesser number of fungal pathogens of canker and dieback disease were known to infect golden rain trees.

During the research conducted in 2019 and 2020, several diseased golden rain tree samples showing branch canker and dieback were collected for disease diagnosis. Although several putative fungal pathogens were reported from these cankers in previous studies, little is known about the aetiology and distribution of canker diseases affecting golden rain trees in China. Therefore, this study was conducted to (i) identify fungal species associated with golden rain tree canker diseases, and (ii) determine the pathogenicity of various fungal species isolated from golden rain tree cankers in Beijing, China.

## Materials and methods

### Sampling and isolation

Researches during 2019 and 2020 were conducted in greenbelts and nurseries in Jiufeng National Forest Park, Haidian Park (Haidian District), Olympic Forest Park (Chaoyang District), and their surrounding areas where golden rain trees planted in Beijing, China. Approximately 10–25 golden rain trees were sampled from each site and cankered tissues from a single branch were collected from each tree showing symptoms typical of branch canker and dieback for detailed examination and fungal isolation. In total, 58 samples were collected. Isolates were obtained by removing spore masses in ascomata/conidiomata onto the surfaces of potato dextrose agar (PDA) and incubated in the laboratory at 25°C for up to 48 h in darkness. Single hyphal tips were transferred to new PDA plates until they were large enough to be used in DNA extraction. Specimens and cultures are deposited at the Museum of Beijing Forestry University (BJFC) and at the working Collection of X.L. Fan (CF) housed at the Beijing Forestry University. All living cultures collected in this study are deposited at the China Forestry Culture Collection Centre (CFCC).

### DNA extraction, amplification, and sequencing

Fungal mycelium for DNA extraction was collected from pure fungal cultures. Genomic DNA was extracted using the modified CTAB method (Doyle and Doyle 1990). PCR amplifications were performed to amplify gene regions of internal transcribed spacer (ITS), actin (*act*), RNA polymerase II subunit (*rpb2*), translation elongation factor 1-alpha (*tef1-α*), and beta-tubulin (*tub2*) using primers as shown in Table S1. Amplification products were electrophoresed in 1% agarose gel and PCR amplicons were sequenced with both forward and reverse primers using an Applied Biosystems 3730xl DNA analyser (Thermo Fisher Scientific, Foster City, CA). DNA sequences generated by each primer combination were used to obtain consensus sequences using Seqman v. 7.1.0 in the DNASTAR lasergene core suite software (DNASTAR Inc., Madison, WI, USA).

### Phylogenetic analyses

The preliminary identities of isolates were carried-out by nucleotide BLAST search performed with ITS sequence against the NCBI database. The ITS sequences generated in this study with high similarity with reference sequences for *Allocryptovalsa, Botryosphaeria, Cytospora, Dothiorella, Eutypella*, and *Peroneutypa* were selected for further study. To clarify the phylogenetic position, the alignments based on different gene regions sequences were performed to compare with other available species in *Botryosphaeria, Dothiorella* (ITS, *tef1-α*, and *tub2), Cytospora* (ITS, *act, rpb2, tef1-α*, and *tub2*), and Diatrypaceae (ITS and *tub2*) obtained from GenBank (Table S2–5), respectively. For individual datasets, sequences were aligned with MAFFT v. 6 (Katoh and Standley 2013) and MEGA v. 6.0 (Tamura et al. 2013). Ambiguously aligned regions were excluded from analyses. Phylogenetic analyses were carried out with Maximum Parsimony (MP), Maximum Likelihood (ML), and Bayesian Inference (BI) analyses. Phylograms were viewed in Figtree v. 1.3.1 (Rambaut and Drummond 2010). Sequences were deposited in GenBank.

### Morphological identification and characterisation

Macroscopic characters of ascomata/condiomata formed on the bark or PDA were observed under the Leica stereomicroscope (M205). Images of conidiogenous cells, conidiophores, conidia, asci, and ascospores were captured using a Nikon Eclipse 80i microscope equipped with a Nikon digital sight DS-Ri2 high definition colour camera with differential interference contrast (DIC). Cultural characteristics of cultures incubated on PDA at 25°C in darkness were recorded after 7 and 14 days according to the colour charts of Rayner (1970). Adobe Photoshop CS v. 5 was used for manual editing.

### Pathogenicity test

The isolates representing different fungal species were selected for inoculations. Asymptomatic 1-year-old healthy plants of golden rain trees grown on 1.3 m high and 1.5 cm thick were surface-sterilised and wounded using a 5-mm-diameter sterilised cork borer. Same-sized agar discs were removed from the actively growing margins of cultures and inserted into the wounds, sealing with moistened cotton wool and protecting with parafilm. Six replicates were conducted for each isolate. Non-colonised PDA agar plugs served as the negative control. After a week, the parafilm and cotton wool were removed. These inoculated plants were maintained in the field. After one month, all the replicates were examined for disease to compare the aggressiveness of the various isolates. After removing the bark, the length of wood discoloration was measured upwards and downwards from the point of inoculation using a digital caliper. Re-isolation was conducted for all experimental groups and control groups by cutting small pieces of discoloured xylem and placing them onto the PDA plates, which identity was verified by morphological and DNA sequence comparisons with the original isolates to fulfill Koch’s postulates. Differences in lesion length between isolates were analysed by one-way analysis of variance (ANOVA) followed by least significant difference (LSD) tests. Statistical analysis was carried out by SPSS v. 20.0 and considered as significant at *p* < 0.05.

## Results

### Fungal isolation

In this study, the sampling focused on symptomatic plants of golden rain trees collected in three parks and their surrounding areas in Beijing, China (Table 1). It is worth mentioning that the appearance of these canker diseases mostly occurs on golden rain trees with sunburns, mechanical injuries, or other forms of wounds. The most common symptoms were irregular wood necrosis and black spots.

**Table 1.** Percent (%) recovery of six fungal species in three sites in Beijing, China.

Locality (GPS Coordinates)	Branch cankers^a^	Number (%) of branch cankers yielding^b^					
		*A. castaneicola*	*B. dothidea*	*C. koelreutericola*	*D. acericola*	*E. citricola*	*P. scoparia*
Jiufeng National Forest Park (40°3ʹ41”N, 116°5ʹ10”E)	22	0 (0)	7 (32)	0 (0)	6 (27)	9 (41)	0 (0)
Haidian Park (39°59ʹ32”N, 116°18ʹ8”E)	25	7 (28)	9 (36)	9 (36)	0 (0)	0 (0)	0 (0)
Olympic Forest Park (40°1ʹ30”N, 116°23ʹ48”E)	11	2 (18)	5 (46)	0 (0)	0 (0)	0 (0)	4 (36)
Total number	58	9 (16)	21 (36)	9 (16)	6 (10)	9 (16)	4 (7)

^a^Number of branch cankers collected from golden rain trees in three sites.

^b^Branch samples per park yielding indicated fungi.

**Figure 1.**
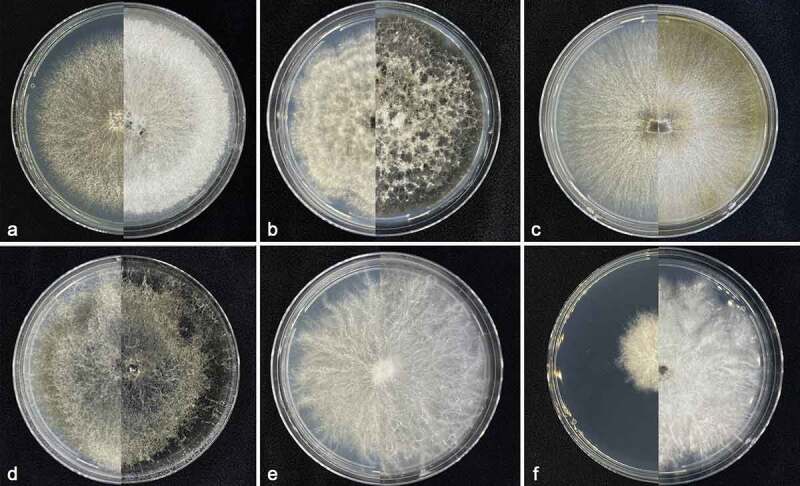
Colonies on PDA at 7 days (left) and 14 days (right). a. *Allocryptovalsa castaneicola* CFCC 56962. b. *Botryosphaeria dothidea* CFCC 56958. c. *Cytospora koelreutericola* CFCC 56961. d. *Dothiorella acericola* CFCC 56966. e. *Eutypella citricola* CFCC 56968. f. *Peroneutypa scoparia* CFCC 56957.

According to ascomata/conidiomata (see below) and colony (Figure 1) morphology and phylogenetic analyses, 64 isolates isolated from 58 samples were identified as *Allocryptovalsa castaneicola* (11 isolates), *Botryosphaeria dothidea* (21 isolates), *Cytospora koelreutericola* (9 isolates), *Dothiorella acericola* (6 isolates), *Eutypella citricola* (9 isolates), and *Peroneutypa scoparia* (8 isolates) based on comparison with the previous morphological descriptions and DNA sequences (Slippers et al. 2004; Carmarán et al. 2006; Trouillas et al. 2011; Phookamsak et al. 2019; Fan et al. 2020; Zhu et al. 2021).

Among these isolates, Diatrypaceae occurring on golden rain trees has the largest number of fungal species, including *A. castaneicola, E. citricola*, and *P. scoparia*. However, species of Botryosphaeriaceae (*B. dothidea* and *D. acericola*) are more dominant in quantity, which were recovered from 36 and 10%, respectively, of necrotic tissues collected from branches. Among them, *B. dothidea* was detected in all sites sampled, and it was also the most frequently isolated in Haidian Park and Olympic Forest Park recovered from 36 and 46%, respectively, of necrotic tissues collected from branches (Table 1).

### Phylogeny

For isolates with identical sequences, only 2–4 representatives were selected from each genus for morphological and phylogenetic analyses. For *Botryosphaeria*, four isolates were sequenced for ITS, *tef1-α*, and *tub2* gene. The combined matrix of *Botryosphaeria* comprised 31 sequences with *Neofusicoccum lutea* (CBS 110497 and CBS 110299) as outgroup, comprising 1299 characters including gaps, of which 1099 characters were constant, 5 variable characters were parsimony-uninformative, and 195 characters were variable and parsimony-informative. MP analysis produced five parsimonious trees (TL = 237, CI = 0.907, RI = 0.940, RC = 0.853). The combined matrix of *Dothiorella* comprised 60 sequences with *N. luteum* (CBS 110497 and CBS 110299) as outgroup, comprising 1341 characters including gaps, of which 957 characters were constant, 54 variable characters were parsimony-uninformative, and 330 characters were variable and parsimony-informative. MP analysis produced two parsimonious trees (TL = 1043, CI = 0.540, RI = 0.848, RC = 0.458). All trees from the ML and Bayesian analyses were identical to that of the MP tree shown. The phylogenetic analyses revealed that six isolates were assigned to two known species, *B. dothidea* (Figure S1) and *D. acericola* (Figure S2).

For *Cytospora*, the combined matrix of ITS, *act, rpb2, tef1-α*, and *tub2* gene included 242 ingroup and the outgroup *Diaporthe vaccinii* CBS 160.32, comprising 3155 characters including gaps (1526 characters were constant, 171 variable characters were parsimony-uninformative, and 1458 characters were variable and parsimony-informative). MP analysis produced 200 parsimonious trees (TL = 9835, CI = 0.305, RI = 0.825, RC = 0.252). All trees from the ML and Bayesian analyses were identical to that of the MP tree shown. The phylogenetic analysis revealed that three isolates were classified as one new species, *C. koelreutericola*, with high support value (MP/ML/BI = 100/100/1) (Figure S3).

For Diatrypaceae, the combined matrix of ITS and *tub* gene included 153 Diatrypaceae sequences and the outgroup *Xylaria hypoxylon* CBS 122620, which comprises 1136 characters including gaps (423 characters were constant, 160 variable characters were parsimony-uninformative, and 553 characters were variable and parsimony-informative). MP analysis produced 200 parsimonious trees (TL = 3786, CI = 0.364, RI = 0.788, RC = 0.287). All trees from the ML and Bayesian analyses were identical to that of the MP tree shown. The phylogenetic analysis revealed that three isolates clustered strongly (MP/ML/BI = 100/99/1) with the ex-type of *A. castaneicola* CFCC 52432, two isolates were identified as *E. citricola* (MP/ML/BI = 100/100/1), another two isolates clustered strongly (MP/ML/BI = 100/100/1) with *P. scoparia* (Figure S4).

### Taxonomy

**Botryosphaeriaceae, Botryosphaeriales**

**Figure 2.**
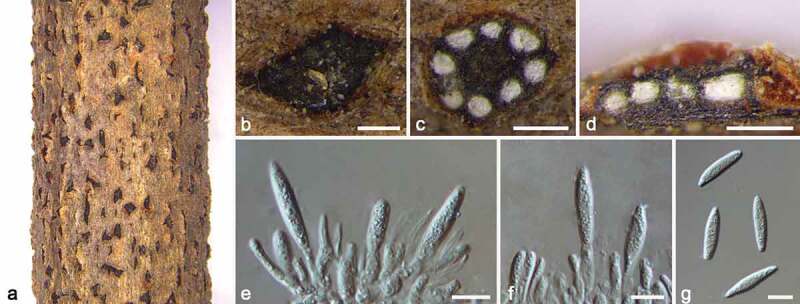
*Botryosphaeria dothidea* (BJFC CF20220201). a–b. Habit of conidiomata on twig. c. Transverse section of conidioma. d. Longitudinal section through conidioma. e–f. Conidiophores and conidiogenous cells. g. Conidia. Scale bars: b–d = 250 μm, e–g = 10 μm.

***Botryosphaeria dothidea*** (Moug.) Ces. & De Not., Comm. Soc. Crittog. Ital. 1: 212, 1863. Figure 2

Synonyms: *Sphaeria dothidea* Moug. Syst. Mycol. 2(2): 423, 1823.

*Descriptions*: see Slippers et al. (2004).

#### Materials examined

CHINA, Beijing, Haidian District, Haidian Park, 116°17′45″E, 39°59′58″N, isolated from branches of *Koelreuteria paniculata*, Yifeng Wang & Hong Gao, 20 April 2019 (BJFC CF20220201; living culture, CFCC 56958); ibid. BJFC CF20220202, living culture CFCC 56959. Beijing, Chaoyang District, Olympic Forest Park, 116°23′33″E, 40°1′47″N, isolated from branches of *Koelreuteria paniculata*, Yifeng Wang & Hong Gao, 20 April 2020 (BJFC CF20220203; living culture, CFCC 56960). Beijing, Haidian District, Jiufeng National Forest Park, 116°4′53″E, 40°4′12″N, isolated from branches of *Koelreuteria paniculata*, Yifeng Wang & Ziqi Pei, 23 April 2020 (BJFC CF20220204; living culture, CFCC 56965).

#### Notes

*Botryosphaeria* (Botryosphaeriaceae, Botryosphaeriales) was established by Cesati de and de Notaris (1863) with *B. dothidea* as the type species (Slippers et al. 2004). However, most boundaries of known *Botryosphaeria* species are tentative due to poor condition of multilocus phylogeny (Ariyawansa et al. 2016; Li et al. 2018; Liang et al. 2019; Jiang et al. 2021). Recently, Zhang et al. (2021) well-defined the species boundaries in this genus and provided justification for reducing previously accepted species to synonymy.

*Botryosphaeria dothidea* is a commonly reported species with a wide host range in China (Tai 1979; Wei 1979; Zhuang 2005; Pan et al. 2019), which can cause various diseases such as canker, dieback, leaf spot, and ring rot. Phylogenetically, four our isolates (CFCC 56958, CFCC 56959, CFCC 56960, CFCC 56965) in this study clustered with *Botryosphaeria dothidea*. Morphologically, they are highly similar to *B. dothidea* in having smooth with granular contents, ellipsoid to fusoid conidia with a subtruncate to bluntly rounded base (Zhang et al. 2021). Therefore, we regarded these four isolates as *B. dothidea*.

**Figure 3.**
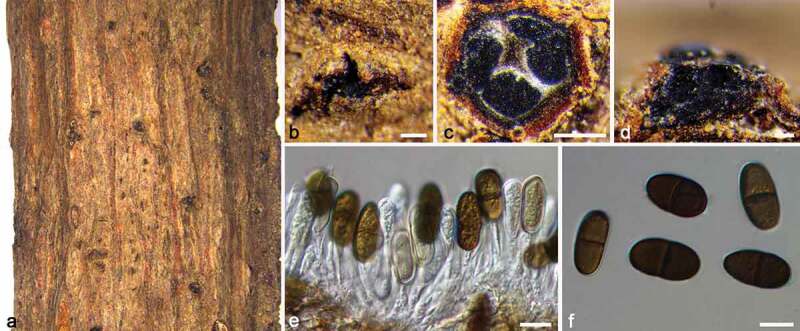
*Dothiorella acericola* (BJFC CF20220208). a–b. Habit of conidiomata on twig. c. Transverse section of conidioma. d. Longitudinal section through conidioma. e. Conidiophores and conidiogenous cells. f. Conidia. Scale bars: b–d = 100, e–f = 10 μm.

***Dothiorella acericola*** Phookamsak, Tennakoon & K.D. Hyde, Fungal Divers. 95: 78, 2019. Figure 3

*Descriptions*: see Phookamsak et al. (2019).

#### Materials examined

CHINA, Beijing, Haidian District, Jiufeng National Forest Park, 116°6′3″E, 40°3′19″N, isolated from branches of *Koelreuteria paniculata*, Yifeng Wang & Ziqi Pei, 23 April 2020 (BJFC CF20220208; living culture, CFCC 56966). ibid. BJFC CF20220209, living culture CFCC 56967.

#### Notes

Based on phylogenetic analyses, our two isolates (CFCC 56966 and CFCC 56967) clustered with the type strains of *D. acericola* (KUMCC 18–0137) with high value (MP/ML/BI = 94/92/1). *Dothiorella acericola* was proposed by Phookamsak et al. (2019), which was isolated from dead hanging twigs of *Acer palmatum* in China. Recent research has shown that jujube trees also were infested by *D. acericola* (Pan et al. 2021b). In this study, we regarded our isolates as *D. acericola* based on the similar morphological characteristics and sustentacular phylogeny and expanded its host range in the current study.

**Cytosporaceae, Diaporthales**

**Figure 4.**
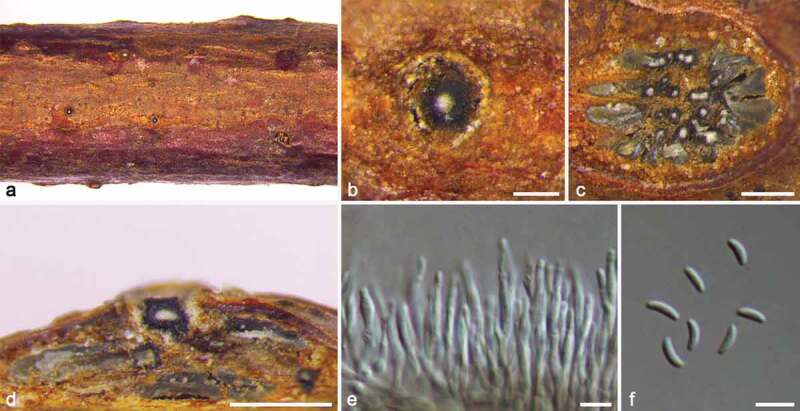
*Cytospora koelreutericola* (BJFC CF20220205). a–b. Habit of conidiomata on twig. c. Transverse section of conidioma. d. Longitudinal section through conidioma. e. Conidiophores and conidiogenous cells. f. Conidia. Scale bars: b = 250 mm, c–d = 500 μm, e–f = 5 μm.

***Cytospora koelreutericola*** M. Pan & X.L. Fan, sp. nov. Figure 4

MycoBank MB 843840

#### Typification

CHINA, Beijing, Haidian District, Haidian Park, 116°18′12″E, 39°59′35″N, isolated from branches of *Koelreuteria paniculata*, Yifeng Wang & Hong Gao, 20 April 2019 (**holotype** BJFC CF20220205; **ex-type culture**, CFCC 56961).

#### Etymology

Named after the host genus on which it was collected, *Koelreuteria*.

#### Descriptions

Sexual morph: Not observed. Asexual morph: Pycnidial stromata ostiolate, immersed in bark, scattered, erumpent through the surface of bark in a large area, with multiple locules. Conceptacle absent. Ectostromatic disc dark grey to black, ovoid to circular, 170–240 µm in diam, with a solitary ostiole per disc. Ostiole buff, above level as the disc surface, 45–90 μm in diam. Locules numerous, subdivided by invaginations with independent walls, 575–1050 µm in diam. Conidiophores hyaline, branched at base or not branched, thin walled, filamentous, 11.5–19.5 × 1–1.5 μm. Conidiogenous cells enteroblastic, phialidic. Conidia hyaline, allantoid, smooth, aseptate, thin-wall, (3.5–)4–5(–5.5) × 1–1.5 (av. = 4.39 ± 0.4 × 1.3 ± 0.2, n = 30) μm.

#### Culture characteristics

Colonies on PDA are initially white and reaching up 40 mm in three days. After a week, colonies become honey and entirely covering the 90 mm Petri dish. Colonies are radial and felty with a uniform texture and deepened gradually in later stage. Pycnidia distributed irregularly on medium surface after 30 days.

#### Additional materials examined

CHINA, Beijing, Haidian District, Haidian Park, 116°18′12″E, 39°59′35″N, isolated from branches of *Koelreuteria paniculata*, Yifeng Wang & Hong Gao, 20 April 2019 (BJFC CF20220206; living culture, CFCC 56970); ibid. BJFC CF2022020, living culture CFCC 56971.

#### Notes

*Cytospora koelreutericola* is associated with canker disease of *Koelreuteria paniculata* in China. Until now, only two *Cytospora* species were recorded to infect *Koelreuteria paniculata* (https://nt.ars-grin.gov/fungaldatabases). Diedicke (1904) provided a description of *C. koelreuteriae* as having 5–6 × 1.5–2 μm conidia. However, this species is lacking detailed descriptions and molecular DNA data. As for *C. koelreuteriae*, it can be easily differentiated from *C. koelreutericola* due to its larger conidia (5–6 × 1.5–2 vs. 4–5 × 1–1.5 μm). *Cytospora leucosperma* was also isolated from golden rain tree in Ukraine (Dudka et al. 2004), but they are phylogenetically distant. In morphology, although *C. koelreutericola* has similar size of conidia to *C. leucosperma*, it can be distinguished by the independent walls of its locules. Phylogenetically, *C. koelreutericola* formed a close group with *C. thailandica*, but differing in its locules arranged circularly, honey coloured colonies, and the larger conidia (4–5 × 1–1.5 vs. 3.8–4 × 1–1.3 μm) (Norphanphoun et al. 2018).

**Diatrypaceae, Xylariales**

**Figure 5.**
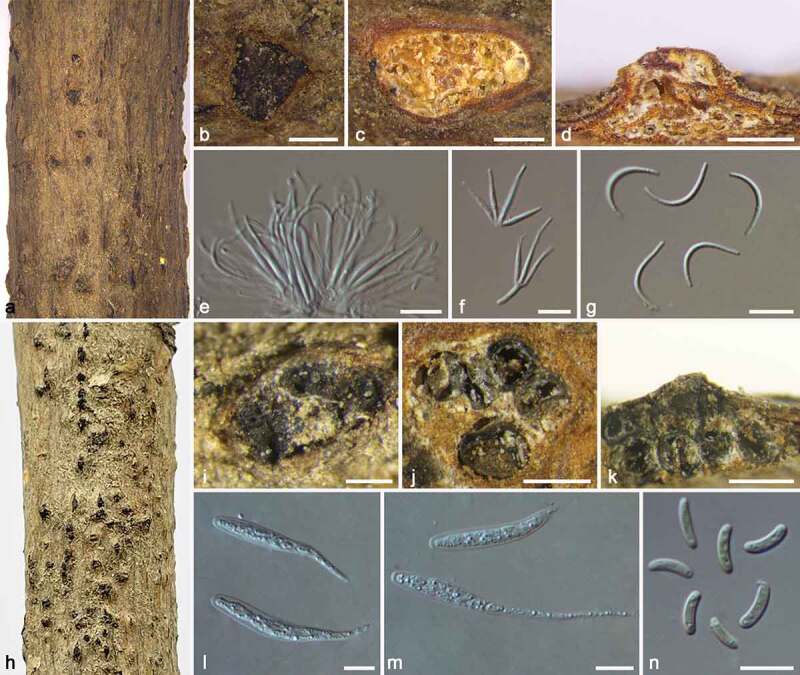
*Allocryptovalsa castaneicola* (BJFC CF20220214). a–b. Habit of conidiomata on twig. c. Transverse section of conidioma. d. Longitudinal section through conidioma. e–f. Conidiophores and conidiogenous cells. g. Conidia. h–i. Habit of ascomata on twig. j. Transverse section of ascoma. k. Longitudinal section through ascoma. l–m. Asci. n. Ascospores. Scale bars: b–d, i–k = 500 μm, e–g, l–n = 10 μm.

***Allocryptovalsa castaneicola*** N. Jiang & X.L. Fan, Front. Microbiol. 12: 646262, 2021. Figure 5

#### Descriptions, emended here

Sexual morph: Ascostromata solitary or gregarious, immersed in the bark, slightly to strongly erumpent through the surface of bark, with 5–10 perithecia arranged irregularly, 1100–2600 µm in diam. Ectostromatic disc grey to black, ovoid to rhombic, with more than five ostioles arranged irregularly per disc, 830–1670 µm in diam. Perithecia dark grey to black, flask-shaped to spherical, 245–430 µm in diam. Asci clavate to elongate obovoid, polysporous, thin-walled, long pedicellate, 35–65 × 5.3–6.5 µm. Ascospores elongate-allantoid, thin-walled, slightly curved, aseptate, 7.5–9.5(–10) × 2–3 µm (av. = 8.94 ± 1.5 × 2.42 ± 0.5, n = 30) µm. Asexual morph: Pycnidial stromata immersed in the bark, scattered, erumpent slightly through the surface of bark. Ectostromatic disc dark brown to black, ovoid to rhombic, 640–1035 µm in diam, with black and inconspicuousa ostiole. Locules numerous, ochreous, circular to ovoid, 790–1560 mm in diam. Conidiogenous cells approximately cylindrical, mostly straight, thin-walled, with wide base producing conidia at the apex, 14.5–25.5 × 1.5–2 µm. Conidia hyaline, filiform, smooth or rough, aseptate, (11.5–)12.5–15.5 × 1–1.5 µm (av. = 14 ± 1.5 × 1.3 ± 0.2, n = 30) µm.

#### Materials examined

CHINA, Beijing, Haidian District, Haidian Park, 116°18′4″E, 39°59′33″N, isolated from branches of *Koelreuteria paniculata*, Yifeng Wang & Hong Gao, 20 April 2019 (BJFC CF20220214; living culture, CFCC 56962); ibid. BJFC CF20220215, living culture CFCC 56963. Beijing, Haidian District, Jiufeng National Forest Park, 116°5′34″E, 40°4′7″N, isolated from branches of *Koelreuteria paniculata*, Yifeng Wang & Ziqi Pei, 23 April 2019 (BJFC CF20220216; living culture, CFCC 56969).

#### Notes

The genus *Allocryptovalsa* was established by Senwanna et al. (2017) with *A. polyspora* as the generic type, which was with the character of immersed perithecia, polysporous asci, and allantoid ascospores. Subsequently, Konta et al. (2020), Hyde et al. (2020), and Zhu et al. (2021) proposed four *Allocryptovalsa* species from *Elaeis guineensis* and *Castanea mollissima*, respectively. In this study, three isolates (CFCC 56962, CFCC 56963, CFCC 56969) were isolated from *Koelreuteria paniculata* and regarded as *A. castaneicola* based on morphological characteristics and molecular data. In this study, we observed the asexual morph of this species from *K. paniculata* in China for the first time. In the meanwhile, we also re-examined the type specimen of *A. castaneicola* and re-described the size of its asci and ascospores.

***Eutypella citricola*** Speg., Anal. Mus. nac. Hist. nat. B. Aires 6: 245, 1898.

*Descriptions*: see Trouillas et al. (2011).

#### Materials examined

CHINA, Beijing, Haidian District, Jiufeng National Forest Park, 116°5′34″E, 40°4′7″N, isolated from branches of *Koelreuteria paniculata*, Yifeng Wang & Ziqi Pei, 23 April 2020 (BJFC CF202202010; living culture, CFCC 56968). ibid. BJFC CF202202011, living culture CFCC 56972.

#### Notes

In this study, the phylogenetic inferences showed the isolates clustered with *Eutypella citricola* (MP/ML/BI = 100/100/1) in phylogram. Morphologically, no asci and ascospores were observed due to the premature ascomata. However, conidiomata are randomly distributed on medium surface after 45 days inoculation on PDA at 25°C in darkness. These isolates are with character of 22.5–36.5 × 1.7–2.5 μm conidiophores and hyaline, filiform, smooth or rough, aseptate, 13.5–19 × 1.5–2 μm conidia. The size of conidia is similar to *E. citricola* in previous study (13.5–19 × 1.5–2 vs. 15–20 × 1.5–2 μm) (Trouillas et al. 2011).

***Peroneutypa scoparia*** (Schwein.) Carmarán & A.I. Romero, Fungal Divers. 23: 84, 2006.

*Synonyms: Sphaeria scoparia* Schwein., Schr. naturf. Ges. Leipzig 1: 37, 1822.

#### Descriptions

see Carmarán et al. (2006).

#### Materials examined

CHINA, Beijing, Chaoyang District, Olympic Forest Park, 116°23′34″E, 40°1′23″N, isolated from branches of *Koelreuteria paniculata*, Yifeng Wang & Hong Gao, 20 April 2020 (BJFC CF20220212; living culture, CFCC 56957). ibid. BJFC CF202202013, living culture CFCC 56964.

#### Notes

The genus *Peroneutypa* was established by Berlese (1902), which accommodated three species without designating the type species. However, Rappaz (1987) synonymised *Peroneutypa* species into *Eutypella*. With the increase in number of species, Carmarán et al. (2006) resurrected *Peroneutypa* to accommodate seven species and eight new combinations. The isolates (CFCC 56957 and CFCC 56964) clustered with *Peroneutypa scoparia* CBS 242.87 in a separate lineage with high support value (MP/ML/BI = 100/100/1). Therefore, these two isolates may be regarded as *P. scoparia*.

### Pathogenicity test

**Figure 6.**
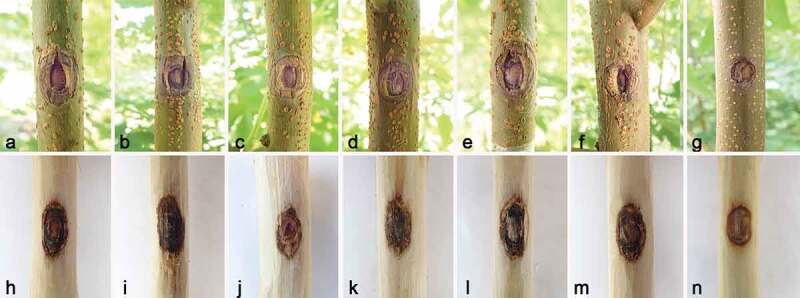
Lesions resulting from inoculation of fungal species onto *Koelreuteria paniculata*, and wound response on the negative control. a, h. *Allocryptovalsa castaneicola*. b, i. *Botryosphaeria dothidea*. c, j. *Cytospora koelreutericola*. d, k. *Dothiorella acericola*. e, l. *Eutypella citricola*. f, m. *Peroneutypa scoparia*. g, n. Negative control. Row 1: lesions on the bark; Row 2: lesions beneath the bark.

Six respective isolates (*Allocryptovalsa castaneicola* CFCC 56962, *Botryosphaeria dothidea* CFCC 56958, *Cytospora koelreutericola* CFCC 56961, *Dothiorella acericola* CFCC 56966, *Eutypella citricola* CFCC 56968, and *Peroneutypa scoparia* CFCC 56957) obtained from golden rain trees were performed for pathogenicity determination using mycelial plug inoculations. After four weeks, all six fungal species produced discoloration on golden rain tree branches (Figure 6). In contrast, there was no lesion development in any of the control inoculations. Isolates were obtained from lesions. Fungal identity was conducted based on morphological comparison with the original isolates and DNA sequence data. As a result, all six tested fungal species were confirmed as causal agents of canker and dieback in golden rain trees.

Among the six species, *B. dothidea* produced the longest lesion on tested branches and caused the lesion lengths ranging from 1.46 to 1.72 cm (mean lesion length = 1.59 cm), followed by *A. castaneicola* which caused the lesion lengths ranging from 1.38 to 1.61 cm (mean lesion length = 1.50 cm). *Dothiorella acericola, E. citricola*, and *P. scoparia* showed similar lesion lengths between each other (Table 2). There is no significant difference between them. The mean lesion length was 1.48, 1.44, and 1.46 cm, respectively. *Cytospora koelreutericola* was significantly lower than other five species in lesion length with canker length averaging 1.36 cm. Fungal recovery varied between 60% and 100% among isolates. ANOVA revealed significant (*p* < 0.05) differences among the treatment means in all six species (Table 2).

**Table 2.** Mean lesion lengths in inoculated golden rain trees, one month after inoculation with six fungal species.

Species	Mean ± SD (cm) ^a^
*Allocryptovalsa castaneicola*	1.50 ± 0.09 b
*Botryosphaeria dothidea*	1.59 ± 0.08 a
*Cytospora koelreutericola*	1.36 ± 0.05 c
*Dothiorella acericola*	1.48 ± 0.07 b
*Eutypella citricola*	1.44 ± 0.09 bc
*Peroneutypa scoparia*	1.46 ± 0.04 b

^a^Different lowercase letters indicate significant differences among six fungal species (LSD test, *p* < 0.05).

## Discussion

In this study, golden rain trees with canker and dieback disease were sampled in Beijing, China. One of the fungi was identified as a new species (*Cytospora koelreutericola*), and five known species (*Allocryptovalsa castaneicola, Botryosphaeria dothidea, Dothiorella acericola, Eutypella citricola, Peroneutypa scoparia*) were first reported from golden rain trees in China. *Allocryptovalsa castaneicola* showed an asexual morph from *Koelreuteria paniculata* in China for the first time. The result of pathogenicity test indicated that all species could cause lesions on golden rain trees. *Botryosphaeria dothidea* appearred to be the most aggressive fungus in this study, followed by *A. castaneicola, D. acericola, P. scoparia, E. citricola*, and *C. koelreutericola*.

This study revealed Diatrypaceae to be the most prevalent canker pathogen of golden rain trees, with three species isolated from symptomatic bark in Beijing, China. Species of Diatrypaceae are frequently saprobic on the decaying wood of trees. However, some species are generally accepted as pathogens associated with plant hosts (Glawe and Rogers 1984). *Allocryptovalsa castaneicola* was isolated from chestnuts in Hebei Province, China (Zhu et al. 2021), however, its pathogenicity to the host was not examined. In this study, the geographical range and host range of *A. castaneicola* was extended, and *A. castaneicola* was regarded as causal agent of canker in golden rain trees. The pathogenicity of *E. citricola* on different hosts is well documented. *Eutypella citricola* has been regarded as a pathogen of branch canker on citrus in Southern California (Mayorquin et al. 2016), grapevine, grapefruit, and orange in Australia (Trouillas et al. 2011; Pitt et al. 2013), apricot and plum in South Africa (Moyo et al. 2018), and pistachio in Iran (Sohrabi et al. 2020). In the current study, *E. citricola* has been found and regarded as a pathogen on golden rain tree in China. *Peroneutypa scoparia* are commonly classified as saprophytes, endophytes, and pathogens on a wide range of woody hosts, which are treated as synonyms of *Eutypa scoparia, Eutypella scoparia, Peroneutypella scoparia, Sphaeria scoparia*, and *Valsa scoparia* (Carmarán et al. 2006). This species has been reported as a pathogen on blueberries in Iran, English walnuts in Czech Republic, and kiwifruit in Chile (Castilla-Cayuman et al. 2018; Eichmeier et al. 2020; Guarnaccia et al. 2020). At present, *P. scoparia* detected during this study in association with losses of golden rain tree was confirmed as pathogenic on this host.

Botryosphaeriaceae species were the second most predominant groups among fungi associated with canker and dieback disease from golden rain trees in this study. Two species of Botryosphaeriaceae were identified: *B. dothidea* and *D. acericola. Botryosphaeria dothidea* has wide host ranges, which is associated with various diseases on over 184 genera (79 families) of plant hosts (Slippers et al. 2004; Chen et al. 2020) and is pathogenic to 50 plant species representing 34 genera and 20 families (Michailides 1991). *Botryosphaeria dothidea* is commonly known as the causal agent of *Botryosphaeria* stem blight in many different woody hosts (Brown-Rytlewski and McManus 2000; Tang et al. 2012; Polashock et al. 2017; Zheng et al. 2020). However, it is worth noting that the pathogenicity of *B. dothidea* differs for strains from different hosts (Latorre and Toledo 1984; Parker and Sutton 1993; Brown-Rytlewski and McManus 2000; Yan et al. 2012; Marsberg et al. 2017). Therefore, variation in pathogenicity among strains of *B. dothidea* from different hosts may exist, but its mechanism remains to be investigated. *Dothiorella* species have been discovered from woody plants in many continents (Phookamsak et al. 2019; Sohrabi et al. 2020; Pan et al. 2021b), however, a minority of pathogenicity tests have ascertained their pathogenicity (Chen et al. 2014; Doll et al. 2015; Lawrence et al. 2017a). In our pathogenicity trials, *D. acericola* caused discolorations that were significantly greater than the controls, suggesting that this species may be classified as a pathogen of golden rain trees.

To our knowledge, the taxonomy of *Cytospora* species have attracted more and more attention (Lawrence et al. 2018; Fan et al. 2020). However, most researches focus on the discovery of new species and new recorded species (Norphanphoun et al. 2017; Pan et al. 2021c), as well as the research on regulator of pathogenicity in important *Cytospora* species (Xiong et al. 2021). Various *Cytospora* species are known as pathogenic fungus from woody plants in different countries. *Cytospora vinacea* and *C. viticola* were reported as pathogenic fungi from grapevine (Lawrence et al. 2017b). Zhou et al. (2020) reported a new pathogen, *C. haidianensis*, on *Euonymus alatus*. The main casual agents of oak canker were determined as *C. quercinum* and *C. vinacea* in Beijing, China (Pan et al. 2021c). In this study, additional canker pathogen isolated in low numbers included *C. koelreutericola* in the family Cytosporaceae. The results indicated that *C. koelreutericola* was less aggressive than other species.

It is interesting to note that the same fungal species may show varying degrees of pathogenicity towards different hosts in recent studies. For example, *E. citricola* caused wood discoloration ranging from 3.7 to 4.33 cm and 1.78 to 2.8 cm after a 5-month incubation period on apricot and plum trees, respectively (Moyo et al. 2018). However, *E. citricola* merely produced lesions of up to 2.89 cm on grapevine after 18 months of incubation in Australia (Pitt et al. 2013). Moreover, *E. citricola* has also been confirmed as pathogens of “Lisbon” lemon because it was capable of producing lesions (less than 2 cm) on shoots after eight months of incubation (Mayorquin et al. 2016). In this study, *E. citricola* was also considered a pathogen on golden rain trees because of the wood discoloration with an average length of 1.44 cm. Although a wide degree of variability in lesion development was found among different plants, we can infer that *E. citricola* was shown the pathogenic potential under field conditions. However, in order to determine host which fungal species was most pathogenic, further research needs to be carried out under identical experimental conditions and inoculation times. Moreover, the results may be different in the detached shoot assays and in the field. We should advocate for the latter, because the detached shoots do not have sufficiently active defence mechanisms.

In summary, this work represented the major research on the identity and pathogenicity of fungal species associated with symptomatic golden rain trees in Beijing, China. Six species were isolated and pathogenicity tests indicated that they exhibited varying degrees of pathogenicity. Two of the pathogenic species, *A. castaneicola* and *D. acericola*, were only based exclusively on taxonomy and phylogeny in previous studies, however, we explored the role of these fungi as canker pathogens in the current study.

The information achieved with this study provided fundamental knowledge to improve disease management. As for canker disease of golden rain trees, future studies should focus on these most widespread and aggressive pathogens reported here, and prevention and control measures should be investigated to mitigate the impact of canker diseases.

## Supplementary Material

Supplemental Material
