# Supplementary material for: Identification and pathogenicity of six fungal species causing canker and dieback disease on golden rain tree in Beijing, China
Source: Mycology. 2022 Jul 5;14(1):37–51. doi: 10.1080/21501203.2022.2096144 (PMC9930857; doi:10.1080/21501203.2022.2096144)
Supplement: Supplemental Material [file TMYC_A_2096144_SM5250.docx]

**Table S1.** Genes used in this study with PCR primers, primer DNA sequence, optimal annealing temperature.

| **Locus** | **Definition** | **Primers** | **Primer DNA sequence (5′**–**3′）** | **Optimal annealing temp (℃)** | **References of primers used** |
| --- | --- | --- | --- | --- | --- |
| ITS | internal transcribed spacer of ribosomal RNA | ITS1 | TCCGTAGGTGAACCTGCGG | 51 | White et al*.* 1990 |
|  |  | ITS4 | TCCTCCGCTTTTGATATGC |  |  |
| *act* | actin | ACT-512F | ATGTGCAAGGCCGGTTTCGC | 61 | Carbone and Kohn, 1999 |
|  |  | ACT-783R | TACGAGTCCTTCTGGCCCAT |  |  |
| *rpb2* | RNA polymerase II second largest subunit | RPB2-5F | GA(T/C)GA(T/C)(A/C)G(A/T)GATCA(T/C)TT(T/C)GG | 52 | Liu et al. 1999 |
|  |  | RPB2-7cR | CCCAT(A/G)GCTTG(T/C)TT(A/G)CCCAT |  |  |
| *tef-1α* | translation elongation factor 1-alpha | EF1-688F | CGGTCACTTGATCTACAAGTGC | 55 | Alves et al. 2008 |
|  |  | EF1-1251R | CCTCGAACTCACCAGTACCG |  |  |
| *tub2* | beta-tubulin | Bt2a | GGTAACCAAATCGGTGCTGCTTTG | 55 | Glass and Donaldson, 1995 |
|  |  | Bt2b | ACCCTCAGTGTAGTGACCCTTGGC |  |  |

Alves A, Crous PW, Correia A, Phillips AJL. 2008. Morphological and molecular data reveal cryptic speciation in *Lasiodiplodia theobromae*. Fungal Diversity. 28:1–13.

Carbone I, Kohn L. 1999. A method for designing primer sets for speciation studies in filamentous ascomycetes. Mycologia. 91:553–556.

Glass NL, Donaldson GC. 1995. Development of primer sets designed for use with the PCR to amplify conserved genes from filamentous ascomycetes. Applied and environmental microbiology. 61:1323–1330.

Liu YL, Whelen S, Hall BD. 1999. Phylogenetic relationships among ascomycetes: evidence from an RNA polymerase II subunit. Molecular Biology and Evolution. 16:1799–1808.

White TJ, Bruns T, Lee S, Taylor J. 1990. Amplification and direct sequencing of fungal ribosomal RNA genes for phylogenetics. PCR Protocols: A Guide to Methods and Applications. 18:315–322.

**Table S2.** Strains of *Botryosphaeria* used in the molecular analyses in this study.

| **Species** | **Strain^1^** | **Host** | **Origin** | **GenBank accession numbers** | | |
| --- | --- | --- | --- | --- | --- | --- |
|  |  |  |  | ITS | *tef1-α* | *tub2* |
| *Botryosphaeria agaves* | MFLUCC 11-0125^T^ | *Agave* sp. | Thailand | JX646791 | JX646856 | JX646841 |
| *Botryosphaeria agaves* | MFLUCC 10-0051 | *Agave* sp. | Thailand | JX646790 | JX646855 | JX646840 |
| *Botryosphaeria corticis* | CBS 119047^T^ | *Vaccinium* sp. | USA | DQ299245 | EU017539 | EU673107 |
| *Botryosphaeria corticis* | ATCC 22927 | *Vaccinium corymbosum* | USA | DQ299247 | EU673291 | EU673108 |
| ***Botryosphaeria dothidea*** | **CFCC 56958** | ***Koelreuteria paniculata*** | **Beijing, China** | **ON376916** | **ON390912** | **ON390921** |
| ***Botryosphaeria dothidea*** | **CFCC 56959** | ***Koelreuteria paniculata*** | **Beijing, China** | **ON376915** | **ON390911** | **ON390920** |
| ***Botryosphaeria dothidea*** | **CFCC 56960** | ***Koelreuteria paniculata*** | **Beijing, China** | **ON376914** | **ON390910** | **ON390919** |
| ***Botryosphaeria dothidea*** | **CFCC 56965** | ***Koelreuteria paniculata*** | **Beijing, China** | **ON376913** | **ON390909** | **ON390918** |
| *Botryosphaeria dothidea* | CBS 110302 | *Vitis vinifera* | Portugal | AY259092 | AY573218 | EU673106 |
| *Botryosphaeria dothidea* | CMW 8000^T^ | *Prunus* sp | Switzerland | AY236949 | AY236898 | AY236927 |
| *Botryosphaeria dothidea*  (*Botryosphaeria auasmontanum*) | CBS 121769^T^ | *Acacia mellifera* | Namibia | EU101303 | EU101348 | NA |
| *Botryosphaeria dothidea*  (*Botryosphaeria wangensis*) | CERC 2298^T^ | *Cedrus deodara* | China | KX278002 | KX278107 | KX278211 |
| *Botryosphaeria dothidea*  (*Botryosphaeria wangensis*) | CERC 2299 | *Cedrus deodara* | China | KX278003 | KX278108 | KX278212 |
| *Botryosphaeria dothidea*  (*Botryosphaeria sinensis*) | CGMCC 3.17723 | *Morus* sp. | China | KT343254 | KU221233 | KX197107 |
| *Botryosphaeria dothidea*  (*Botryosphaeria sinensis*) | CGMCC 3.17724 | *Juglans regia* | China | KT343256 | KU221234 | KX197108 |
| *Botryosphaeria dothidea*  (*Botryosphaeria minutispermatia*) | GZCC 16-0013^T^ | Dead wood | China | KX447675 | KX447678 | NA |
| *Botryosphaeria dothidea*  (*Botryosphaeria minutispermatia*) | GZCC 16-0014 | Dead wood | China | KX447676 | KX447679 | NA |
| *Botryosphaeria fabicerciana* | CMW 27094^T^ | *Eucalyptus* sp. | China | HQ332197 | HQ332213 | KF779068 |
| *Botryosphaeria fabicerciana* | CMW 27121 | *Eucalyptus* sp. | China | HQ332198 | HQ332214 | KF779069 |
| *Botryosphaeria fabicerciana*  (*Botryosphaeria fusispora*) | MFLUCC 10-0098^T^ | *Entada* sp. | Thailand | JX646789 | JX646854 | JX646839 |
| *Botryosphaeria kuwatsukai* | CBS 135219^T^ | *Malus domestica* | China | KJ433388 | KJ433410 | NA |
| *Botryosphaeria kuwatsukai*  (*Botryosphaeria rosaceae*) | CGMCC 3.18007 | *Malus* sp. | China | KX197074 | KX197094 | KX197101 |
| *Botryosphaeria kuwatsukai*  (*Botryosphaeria rosaceae*) | CGMCC 3.18008^T^ | *Amygdalus* sp. | China | KX197075 | KX197095 | KX197102 |
| *Botryosphaeria kuwatsukai* | LSP 5 | *Pyrus* sp. | China | KJ433395 | KJ433417 | NA |
| *Botryosphaeria qingyuanensis* | CERC 2946^T^ | *Eucalyptus* hybrid | China | KX278000 | KX278105 | KX278209 |
| *Botryosphaeria qingyuanensis* | CERC 2947 | *Eucalyptus* hybrid | China | KX278001 | KX278106 | KX278210 |
| *Botryosphaeria ramosa* | CBS 122069^T^ | *Eucalyptus camaldulensis* | Australia | EU144055 | EU144070 | KF766132 |
| *Botryosphaeria ramosa*  (*Botryosphaeria pseudoramosa*) | CERC 1999 | *Eucalyptus* hybrid | China | KX277988 | KX278093 | KX278197 |
| *Botryosphaeria ramosa*  (*Botryosphaeria pseudoramosa*) | CERC 2001^T^ | *Eucalyptus* hybrid | China | KX277989 | KX278094 | KX278198 |
| *Botryosphaeria scharifii* | IRAN 1543C | *Mangifera indica* | Iran | JQ772019 | JQ772056 | NA |
| *Botryosphaeria scharifii* | IRAN 1529C^T^ | *Mangifera indica* | Iran | JQ772020 | JQ772057 | NA |
| *Neofusicoccum luteum* | CBS 110299 | *Vitis vinifera* | Portugal | AY259091 | AY573217 | NA |
| *Neofusicoccum luteum* | CBS 110497 | *Vitis vinifera* | Portugal | EU673311 | AY573235 | NA |

^1^ Acronyms: CBS: Westerdijk Fungal Biodiversity Institute (CBS-KNAW Fungal Biodiversity Centre), Utrecht, The Netherlands; CERC: Culture collection of China Eucalypt Research Centre, Chinese Academy of Forestry, ZhanJiang, GuangDong, China; CFCC: China Forestry Culture Collection Centre, Beijing, China; CGMCC: China General Microbiological Culture Collection Center; GZCC: Guizhou Academy of Agricultural Sciences Culture Collection, GuiZhou, China; CMW: Culture collection of Michael Wingfield, University of Pretoria, South Africa; IRAN: Iranian Fungal Culture Collection, Iranian Research Institute of Plant Protection, Iran; MFLUCC: Mae Fah Luang University Culture Collection, Thailand; NA: not applicable. All the new isolates used in this study are in bold and the type materials are marked with T.

**Table S3.** Strains of *Dothiorella* used in the molecular analyses in this study.

| **Species** | **Strain^1^** | **Host** | **Origin** | **GenBank accession numbers** | | |
| --- | --- | --- | --- | --- | --- | --- |
|  |  |  |  | ITS | *tef1-α* | *tub2* |
| *Dothiorella acacicola* | CBS 141295^T^ | *Acacia mearnsii* | France | KX228269 | KX228376 | NA |
| *Dothiorella acericola* | KUMCC 18-0137^T^ | *Acer palmatum* | China | MK359449 | MK361182 | NA |
| *Dothiorella acericola* | HNXX032 | *Ziziphus jujuba* | China | KY385661 | KY393212 | KY393178 |
| ***Dothiorella acacicola*** | **CFCC 56966** | ***Koelreuteria paniculata*** | **Beijing, China** | **ON376927** | **ON390917** | **NA** |
| ***Dothiorella acericola*** | **CFCC 56967** | ***Koelreuteria paniculata*** | **Beijing, China** | **ON376928** | **ON390916** | **NA** |
| *Dothiorella alpina* | CGMCC 3.18001^T^ | *Platycladus orientalis* | China | KY385661 | KY393212 | KY393178 |
| *Dothiorella brevicollis* | CBS 130411^T^ | *Acacia karroo* | South Africa | JQ239403 | JQ239390 | JQ239371 |
| *Dothiorella brevicollis* | CMW 36464 | *Acacia karroo* | South Africa | JQ239404 | JQ239391 | JQ239372 |
| *Dothiorella capri-amissi* | CBS 121763^T^ | *Acacia erioloba* | South Africa | EU101323 | EU101368 | KX464850 |
| *Dothiorella capri-amissi* | CMW 25404 | *Acacia erioloba* | South Africa | EU101324 | EU101369 | NA |
| *Dothiorella casuarini* | CBS 120688^T^ | *Casuarina* sp. | Australia | DQ846773 | DQ875331 | NA |
| *Dothiorella casuarini* | CBS 120690 | *Casuarina* sp. | Australia | DQ846774 | DQ875333 | NA |
| *Dothiorella citricola* | CBS 124729^T^ | *Citrus sinensis* | New Zealand | EU673323 | EU673290 | KX464853 |
| *Dothiorella citricola* | CBS 124728 | *Citrus sinensis* | New Zealand | EU673322 | EU673289 | KX464852 |
| *Dothiorella citrimurcotticola* | CGMCC3.20392 | *Citrus unshiu* | China | MW880663 | MW884166 | MW884195 |
| *Dothiorella citrimurcotticola* | CGMCC3.20393 | *Citrus maxima* | China | MW880665 | MW884168 | MW884197 |
| *Dothiorella citrimurcotticola* | CGMCC3.20394^T^ | *Citrus reticulata ×Citrus sinensis* | China | MW880661 | MW884164 | MW884193 |
| *Dothiorella citrimurcotticola* | CGMCC3.20395 | *Citrus reticulata ×Citrus sinensis* | China | MW880662 | MW884165 | MW884194 |
| *Dothiorella citrimurcotticola* | BE6 | *Citrus unshiu* | China | MW880666 | MW884169 | MW884198 |
| *Dothiorella diospyricola* | CBS 145972^T^ | *Diospyros mespiliformis* | South Africa | MT587398 | MT592110 | MT592581 |
| *Dothiorella dulcispinae* | CBS 130413^T^ | *Acacia karroo* | South Africa | JQ239400 | JQ239387 | JQ239373 |
| *Dothiorella dulcispinae* | CMW 36462 | *Acacia karroo* | South Africa | JQ239402 | JQ239389 | JQ239375 |
| *Dothiorella eriobotryae* | CBS 140852^T^ | *Eriobotrya japonica* | Spain | KT240287 | KT240262 | MT592582 |
| *Dothiorella heterophyllae* | CMW46458^T^ | *Acacia heterophylla* | La Réunion | MN103794 | MH548348 | MH548324 |
| *Dothiorella iranica* | CBS 124722^T^ | *Olea europea* | Iran | KC898231 | KC898214 | KX464856 |
| *Dothiorella iranica* | MFLUCC 15-0656 | *Olea europea* | Iran | KX765302 | KX765303 | NA |
| *Dothiorella koae* | CMW 48017 | *Paliurus* | La Réunion | MH447652 | MH548338 | MH548327 |
| *Dothiorella lampangensis* | MFLUCC 18-0232^T^ | *Rutaceae* | Thailand | MK347758 | MK340869 | MK412874 |
| *Dothiorella longicollis* | CBS 122068^T^ | *Lysiphyllum cunninghamii* | Australia | EU144054 | EU144069 | KF766130 |
| *Dothiorella longicollis* | CBS 122066 | *Terminalia* sp. | Australia | EU144052 | EU144067 | KX464857 |
| *Dothiorella magnoliae* | CFCC 51563^T^ | *Magnolia grandiflora* | China | KY111247 | KY213686 | NA |
| *Dothiorella magnoliae* | CFCC 51564 | *Magnolia grandiflora* | China | KY111248 | KY213687 | NA |
| *Dothiorella mangifericola* | CBS 124727^T^ | *Mangifera indica* | Iran | KC898221 | KC898204 | NA |
| *Dothiorella mangifericola* | IRAN 1545C | *Mangifera indica* | Iran | KC898223 | KC898206 | NA |
| *Dothiorella moneti* | MUCC 505^T^ | *Acacia rostellifera* | Australia | EF591920 | EF591971 | EF591954 |
| *Dothiorella moneti* | MUCC 507 | *Acacia rostellifera* | Australia | EF591922 | EF591973 | EF591956 |
| *Dothiorella plurivora* | CBS 124725 | *Prunus armeniaca* | Iran | KC898225 | KC898213 | KX464875 |
| *Dothiorella pretoriensis* | CBS 130404^T^ | *Acacia karroo* | South Africa | JQ239405 | JQ239392 | JQ239376 |
| *Dothiorella pretoriensis* | CMW 36481 | *Acacia karroo* | South Africa | JQ239406 | JQ239393 | JQ239377 |
| *Dothiorella prunicola* | CBS 124723^T^ | *Prunus dulcis* | Portugal | EU673313 | EU673280 | NA |
| *Dothiorella reunionis* | CMW46457 | *Acacia heterophylla* | La Réunion | MH447649 | MH548347 | NA |
| *Dothiorella santali* | MUCC 509^T^ | *Santalum acuminatum* | Australia | EF591924 | EF591975 | EF591958 |
| *Dothiorella santali* | MUCC 508 | *Santalum acuminatum* | Australia | EF591923 | EF591974 | EF591957 |
| *Dothiorella sarmentorum* | IMI 63581b^T^ | *Ulmus* sp. | England | AY573212 | AY573235 | NA |
| *Dothiorella sarmentorum* | CBS 115038 | *Malus pumila* | Netherlands | AY573206 | AY573223 | EU673101 |
| *Dothiorella* sp. 1 | CBS 121783 | *Acacia mearnsii* | South Africa | EU101333 | EU101378 | KX464859 |
| *Dothiorella* sp. 1 | CBS 121784 | *Acacia mearnsii* | South Africa | EU101331 | EU101376 | KX464860 |
| *Dothiorella striata* | CBS 124731^T^ | *Citrus sinensis* | New Zealand | EU673321 | EU673288 | EU673143 |
| *Dothiorella striata* | CBS 124730 | *Citrus sinensis* | New Zealand | EU673320 | EU673287 | EU673142 |
| *Dothiorella tectonae* | MFLUCC 12-0382^T^ | *Tectona grandis* | Thailand | KM396899 | KM409637 | KM510357 |
| *Dothiorella thailandica* | MFLUCC 11-0438^T^ | *Bamboo culm* | Thailand | JX646796 | JX646861 | JX646844 |
| *Dothiorella thripsita* | CBS 125445^T^ | *Acacia harpophylla* | Australia | FJ824738 | KJ573639 | KJ577550 |
| *Dothiorella ulmacea* | CBS 138855^T^ | *Ulmus laevis* | Germany | KR611881 | KR611910 | KR611909 |
| *Dothiorella ulmacea* | CPC 24945 | *Ulmus laevis* | Germany | KR611882 | KR857697 | NA |
| *Dothiorella uruguayensis* | CBS 124908^T^ | *Hexachlamis edulis* | Uruguay | EU080923 | EU863180 | KX464886 |
| *Dothiorella vinea-gemmae* | DAR 81012^T^ | *Vitis vinifera* | Australia | KJ573644 | KJ573641 | NA |
| *Dothiorella viticola* | CBS 117009^T^ | *Vitis vinifera* cv. *Garnatxa Negra* | Spain | AY905554 | AY905559 | EU673104 |
| *Dothiorella viticola* | GAR09 | *Vitis* sp. | French | KT595694 | KX098285 | KT595695 |
| *Dothiorella yunnana* | CGMCC 3.17999^T^ | *Camellia* sp. | China | KX499643 | KX499649 | NA |
| *Dothiorella yunnana* | CGMCC 3.18000 | *Camellia* sp. | China | KX499644 | KX499650 | NA |
| *Neofusicoccum luteum* | CBS 110299 | *Vitis vinifera* | Portugal | AY259091 | AY573217 | NA |
| *Neofusicoccum luteum* | CBS 110497 | *Vitis vinifera* | Portugal | EU673311 | AY573235 | NA |

^1^ Acronyms: CBS: Westerdijk Fungal Biodiversity Institute (CBS-KNAW Fungal Biodiversity Centre), Utrecht, The Netherlands; CFCC: China Forestry Culture Collection Centre, Beijing, China; CMW: Culture collection of Michael Wingfield, University of Pretoria, South Africa; CPC: Culture collection of Pedro Crous, The Netherlands; IMI: Culture collection of the International Mycological Institute, CABI Bioscience, Egham, Surrey, UK; IRAN: Iranian Fungal Culture Collection, Iranian Research Institute of Plant Protection, Iran; MFLUCC: Mae Fah Luang University Culture Collection, Thailand; MUCC: Murdoch University Culture Collection, Perth, Australia; NA: not applicable. All the new isolates used in this study are in bold and the type materials are marked with T.

**Table S4.** Strains of *Cytospora* used in the molecular analyses in this study.

| **Species** | **Strain^1^** | **Host** | **Origin** | **GenBank accession numbers** | | | | |
| --- | --- | --- | --- | --- | --- | --- | --- | --- |
|  |  |  |  | ITS | *act* | *rpb2* | *tef1-α* | *tub2* |
| *Cytospora ailanthicola* | CFCC 89970 | *Ailanthus altissima* | Ningxia, China | MH933618 | MH933526 | MH933592 | MH933494 | MH933565 |
| *Cytospora albodisca* | CFCC 53161 | *Platycladus orientalis* | Beijing, China | MW418406 | MW422899 | MW422909 | MW422921 | MW422933 |
| *Cytospora albodisca* | CFCC 54373 | *Platycladus orientalis* | Beijing, China | MW418407 | MW422900 | MW422910 | MW422922 | MW422934 |
| *Cytospora ampulliformis* | MFLUCC 16-0583^T^ | *Sorbus intermedia* | Russia | KY417726 | KY417692 | KY417794 | NA | NA |
| *Cytospora ampulliformis* | MFLUCC 16-0629 | *Acer platanoides* | Russia | KY417727 | KY417693 | KY417795 | NA | NA |
| *Cytospora amygdali* | CBS 144233^T^ | *Prunus dulcis* | California, USA | MG971853 | MG972002 | NA | MG971659 | MG971718 |
| *Cytospora atrocirrhata* | CFCC 89615 | *Juglans regia* | Qinghai, China | KR045618 | KF498673 | KU710946 | KP310858 | KR045659 |
| *Cytospora atrocirrhata* | CFCC 89616 | *Juglans regia* | Qinghai, China | KR045619 | KF498674 | KU710947 | KP310859 | KR045660 |
| *Cytospora atrocirrhata* | CXY 1401 | *Populus* sp. | Inner Mongolia, China | JX534242 | NA | NA | NA | KM034904 |
| *Cytospora atrocirrhata* | CXY 1402 | *Populus* sp. | Inner Mongolia, China | JX534243 | NA | NA | NA | KM034903 |
| *Cytospora beilinensis* | CFCC 50493^T^ | *Pinus armandii* | Beijing, China | MH933619 | MH933527 | NA | MH933495 | MH933561 |
| *Cytospora beilinensis* | CFCC 50494 | *Pinus armandii* | Beijing, China | MH933620 | MH933528 | NA | MH933496 | MH933562 |
| *Cytospora berberidis* | CFCC 89927^T^ | *Berberis dasystachya* | Qinghai, China | KR045620 | KU710990 | KU710948 | KU710913 | KR045661 |
| *Cytospora berberidis* | CFCC 89933 | *Berberis dasystachya* | Qinghai, China | KR045621 | KU710991 | KU710949 | KU710914 | KR045662 |
| *Cytospora bungeana* | CFCC 50495^T^ | *Pinus bungeana* | Shanxi, China | MH933621 | MH933529 | MH933593 | MH933497 | MH933563 |
| *Cytospora bungeana* | CFCC 50496 | *Pinus bungeana* | Shanxi, China | MH933622 | MH933530 | MH933594 | MH933498 | MH933564 |
| *Cytospora californica* | CBS 144234^T^ | *Juglans regia* | California, USA | MG971935 | MG972083 | NA | MG971645 | NA |
| *Cytospora carbonacea* | CFCC 89947 | *Ulmus pumila* | Qinghai, China | KR045622 | KP310842 | KU710950 | KP310855 | KP310825 |
| *Cytospora carpobroti* | CMW 48981^T^ | *Carpobrotus edulis* | South Africa | MH382812 | NA | NA | MH411212 | MH411207 |
| *Cytospora celtidicola* | CFCC 50497^T^ | *Celtis sinensis* | Anhui, China | MH933623 | MH933531 | MH933595 | MH933499 | MH933566 |
| *Cytospora celtidicola* | CFCC 50498 | *Celtis sinensis* | Anhui, China | MH933624 | MH933532 | MH933596 | MH933500 | MH933567 |
| *Cytospora centrivillosa* | MFLUCC 16-1206^T^ | *Sorbus domestica* | Italy | MF190122 | NA | MF377600 | NA | NA |
| *Cytospora centrivillosa* | MFLUCC 17-1660 | *Sorbus domestica* | Italy | MF190123 | NA | MF377601 | NA | NA |
| *Cytospora ceratosperma* | CFCC 89624 | *Juglans regia* | Gansu, China | KR045645 | NA | KU710976 | KP310860 | KR045686 |
| *Cytospora ceratosperma* | CFCC 89625 | *Juglans regia* | Gansu, China | KR045646 | NA | KU710977 | KP31086 | KR045687 |
| *Cytospora ceratospermopsis* | CFCC 89626^T^ | *Juglans regia* | Shaanxi, China | KR045647 | KU711011 | KU710978 | KU710934 | KR045688 |
| *Cytospora ceratospermopsis* | CFCC 89627 | *Juglans regia* | Shaanxi, China | KR045648 | KU711012 | KU710979 | KU710935 | KR045689 |
| *Cytospora chrysosperma* | CFCC 89629 | *Salix psammophila* | Shaanxi, China | KF765673 | NA | KF765705 | NA | NA |
| *Cytospora chrysosperma* | CFCC 89981 | *Populus alba* subsp.  *pyramidalis* | Gansu, China | MH933625 | MH933533 | MH933597 | MH933501 | MH933568 |
| *Cytospora chrysosperma* | CFCC 89982 | *Ulmus pumila* | Tibet, China | KP281261 | KP310835 | NA | KP310848 | KP310818 |
| *Cytospora cinnamomea* | CFCC 53178^T^ | *Prunus armeniaca* | Xinjiang, China | MK673054 | MK673024 | NA | NA | MK672970 |
| *Cytospora coryli* | CFCC 53162^T^ | *Corylus mandshurica* | Beijing, China | MN854450 | NA | MN850751 | MN850758 | MN861120 |
| *Cytospora corylina* | CFCC 54684^T^ | *Corylus heterophylla* | Beijing, China | MW839861 | MW815951 | MW815937 | MW815886 | MW883969 |
| *Cytospora corylina* | CFCC 54685 | *Corylus heterophylla* | Beijing, China | MW839862 | MW815952 | MW815938 | MW815887 | MW883970 |
| *Cytospora corylina* | CFCC 54686 | *Corylus heterophylla* | Beijing, China | MW839863 | MW815953 | MW815939 | MW815888 | MW883971 |
| *Cytospora corylina* | CFCC 54687 | *Corylus heterophylla* | Beijing, China | MW839864 | MW815954 | MW815940 | MW815889 | MW883972 |
| *Cytospora cotini* | MFLUCC 14-1050^T^ | *Cotinus coggygria* | Russia | KX430142 | NA | KX430144 | NA | NA |
| *Cytospora cotoneastricola* | CF 20197027 | *Cotoneaster* sp. | Tibet, China | MK673072 | MK673042 | MK673012 | MK672958 | MK672988 |
| *Cytospora cotoneastricola* | CF 20197028 | *Cotoneaster* sp. | Tibet, China | MK673073 | MK673043 | MK673013 | MK672959 | MK672989 |
| *Cytospora cotoneastricola* | CF 20197030 | *Cotoneaster* sp. | Tibet, China | MK673074 | MK673044 | MK673014 | MK672960 | MK672990 |
| *Cytospora cotoneastricola* | CF 20197031^T^ | *Cotoneaster* sp. | Tibet, China | MK673075 | MK673045 | MK673015 | MK672961 | MK672991 |
| *Cytospora curvata* | MFLUCC 15-0865^T^ | *Salix alba* | Russia | KY417728 | KY417694 | KY417796 | NA | NA |
| *Cytospora curvispora* | CFCC 54000^T^ | *Corylus heterophylla* | Beijing, China | MW839851 | MW815931 | MW815945 | MW815880 | MW883963 |
| *Cytospora curvispora* | CFCC 54001 | *Corylus heterophylla* | Beijing, China | MW839853 | MW815932 | MW815946 | MW815881 | MW883964 |
| *Cytospora curvispora* | CFCC 54676 | *Corylus heterophylla* | Beijing, China | MW839854 | MW815933 | MW815947 | MW815882 | MW883965 |
| *Cytospora curvispora* | CFCC 54677 | *Corylus heterophylla* | Beijing, China | MW839855 | MW815934 | MW815948 | MW815883 | MW883966 |
| *Cytospora curvispora* | CFCC 54678 | *Corylus heterophylla* | Beijing, China | MW839856 | MW815935 | MW815949 | MW815884 | MW883967 |
| *Cytospora curvispora* | CFCC 54679 | *Corylus heterophylla* | Beijing, China | MW839857 | MW815936 | MW815950 | MW815885 | MW883968 |
| *Cytospora davidiana* | CXY 1350^T^ | *Populus davidiana* | Inner Mongolia,  China | KM034870 | NA | NA | NA | NA |
| *Cytospora discotoma* | CFCC 53137 ^T^ | *Platycladus orientalis* | Beijing, China | MW418404 | MW422897 | MW422907 | MW422919 | MW422931 |
| *Cytospora discotoma* | CFCC 54368 | *Platycladus orientalis* | Beijing, China | MW418405 | MW422898 | MW422908 | MW422920 | MW422932 |
| *Cytospora leucostoma* | MFLUCC 15-0864 | *Crataegus monogyna* | Russia | KY417729 | KY417695 | KY417797 | NA | NA |
| *Cytospora leucostoma* | MFLUCC 16-0574 | *Crataegus monogyna* | Russia | KY417731 | KY417697 | KY417799 | NA | NA |
| *Cytospora donglingensis* | CFCC 53159 ^T^ | *Platycladus orientalis* | Beijing, China | MW418412 | MW422903 | MW422915 | MW422927 | MW422939 |
| *Cytospora donglingensis* | CFCC 53160 | *Platycladus orientalis* | Beijing, China | MW418414 | MW422905 | MW422917 | MW422929 | MW422941 |
| *Cytospora donglingensis* | CFCC 54371 | *Platycladus orientalis* | Beijing, China | MW418413 | MW422904 | MW422916 | MW422928 | MW422940 |
| *Cytospora donglingensis* | CFCC 54372 | *Platycladus orientalis* | Beijing, China | MW418415 | MW422906 | MW422918 | MW422930 | MW422942 |
| *Cytospora elaeagni* | CFCC 89632 | *Elaeagnus angustifolia* | Ningxia, China | KR045626 | KU710995 | KU710955 | KU710918 | KR045667 |
| *Cytospora elaeagni* | CFCC 89633 | *Elaeagnus angustifolia* | Ningxia, China | KF765677 | KU710996 | KU710956 | KU710919 | KR045668 |
| *Cytospora elaeagnicola* | CFCC 52882^T^ | *Elaeagnus angustifolia* | China | MK732341 | MK732344 | MK732347 | NA | NA |
| *Cytospora elaeagnicola* | CFCC 52883 | *Elaeagnus angustifolia* | China | MK732342 | MK732345 | MK732348 | NA | NA |
| *Cytospora elaeagnicola* | CFCC 52884 | *Elaeagnus angustifolia* | China | MK732343 | MK732346 | MK732349 | NA | NA |
| *Cytospora erumpens* | CFCC 50022 | *Prunus padus* | Shanxi, China | MH933627 | MH933534 | NA | MH933502 | MH933569 |
| *Cytospora erumpens* | MFLUCC 16-0580^T^ | *Salix* × *fragilis* | Russia | KY417733 | KY417699 | KY417801 | NA | NA |
| *Cytospora erumpens* | CFCC 53163 | *Prunus padus* | Xinjiang, China | MK673059 | MK673029 | MK673000 | MK672948 | MK672975 |
| *Cytospora eucalypti* | CBS 144241 | *Eucalyptus globulus* | California, USA | MG971907 | MG972056 | NA | MG971617 | MG971772 |
| *Cytospora euonymicola* | CFCC 50499^T^ | *Euonymus kiautschovicus* | Shaanxi, China | MH933628 | MH933535 | MH933598 | MH933503 | MH933570 |
| *Cytospora euonymicola* | CFCC 50500 | *Euonymus kiautschovicus* | Shaanxi, China | MH933629 | MH933536 | MH933599 | MH933504 | MH933571 |
| *Cytospora euonymina* | CFCC 89993^T^ | *Euonymus kiautschovicus* | Shanxi, China | MH933630 | MH933537 | MH933600 | MH933505 | MH933590 |
| *Cytospora euonymina* | CFCC 89999 | *Euonymus kiautschovicus* | Shanxi, China | MH933631 | MH933538 | MH933601 | MH933506 | MH933591 |
| *Cytospora fraxinigena* | MFLU 17-0880 | *Fraxinus ornus* | NA | MF190133 | NA | NA | NA | NA |
| *Cytospora fraxinigena* | MFLUCC 14-0868^T^ | *Fraxinus ornus* | Italy | MF190133 | NA | NA | NA | NA |
| *Cytospora fugax* | CXY 1371 | *Populus simonii* | Jilin, China | KM034852 | NA | NA | NA | KM034891 |
| *Cytospora fugax* | CXY 1381 | *Populus ussuriensis* | Heilongjiang, China | KM034853 | NA | NA | NA | KM034890 |
| *Cytospora fusispora* | NFCCI 4372 | NA | India | MN227694 | NA | NA | NA | NA |
| *Cytospora galegicola* | MFLUCC 18-1199^T^ | *Galega officinalis* | Forlì-Cesena, Italy | MK912128 | MN685810 | MN685820 | NA | NA |
| *Cytospora gigalocus* | CFCC 89620^T^ | *Juglans regia* | Qinghai, China | KR045628 | KU710997 | KU710957 | KU710920 | KR045669 |
| *Cytospora gigalocus* | CFCC 89621 | *Juglans regia* | Qinghai, China | KR045629 | KU710998 | KU710958 | KU710921 | KR045670 |
| *Cytospora gigaspora* | CFCC 50014 | *Juniperus procumbens* | Shanxi, China | KR045630 | KU710999. | KU710959 | KU710922 | KR045671 |
| *Cytospora gigaspora* | CFCC 89634^T^ | *Salix psammophila* | Shaanxi, China | KF765671 | KU711000 | KU710960 | KU710923 | KR045672 |
| *Cytospora globosa* | MFLU 16-2054^T^ | *Abies alba* | Italy | MT177935 | NA | MT432212 | MT454016 | NA |
| *Cytospora granati* | CBS 144237^T^ | *Punica granatum* | California, USA | MG971799 | MG971949 | NA | MG971514 | MG971664 |
| *Cytospora haidianensis* | CFCC 54056 | *Euonymus alatus* | Beijing, China | MT360041 | MT363978 | MT363987 | MT363997 | MT364007 |
| *Cytospora haidianensis* | CFCC 54057^T^ | *Euonymus alatus* | Beijing, China | MT360042 | MT363979 | MT363988 | MT363998 | MT364008 |
| *Cytospora haidianensis* | CFCC 54184 | *Euonymus alatus* | Beijing, China | MT360043 | MT363980 | MT363989 | MT363999 | MT364009 |
| *Cytospora hippophaës* | CFCC 89639 | *Hippophaë rhamnoides* | Gansu, China | KR045632 | KU711001 | KU710961 | KU710924 | KR045673 |
| *Cytospora hippophaës* | CFCC 89640 | *Hippophaë rhamnoides* | Gansu, China | KF765682 | KF765730 | KU710962 | KP310865 | KR045674 |
| *Cytospora japonica* | CFCC 89956 | *Prunus cerasifera* | Ningxia, China | KR045624 | KU710993 | KU710953 | KU710916 | KR045665 |
| *Cytospora japonica* | CFCC 89960 | *Prunus cerasifera* | Ningxia, China | KR045625 | KU710994 | KU710954 | KU710917 | KR045666 |
| *Cytospora joaquinensis* | CBS 144235 | *Populus deltoides* | California, USA | MG971895 | MG972044 | NA | MG971605 | MG971761 |
| *Cytospora junipericola* | BBH 42444 | *Juniperus communis* | Italy | MF190126 | NA | NA | MF377579 | NA |
| *Cytospora junipericola* | MFLU 17-0882^T^ | *Juniperus communis* | Italy | MF190125 | NA | NA | MF377580 | NA |
| *Cytospora juniperina* | CFCC 50501^T^ | *Juniperus przewalskii* | Sichuan, China | MH933632 | MH933539 | MH933602 | MH933507 | NA |
| *Cytospora juniperina* | CFCC 50502 | *Juniperus przewalskii* | Sichuan, China | MH933633 | MH933540 | MH933603 | MH933508 | MH933572 |
| *Cytospora juniperina* | CFCC 50503 | *Juniperus przewalskii* | Sichuan, China | MH933634 | MH933541 | MH933604 | MH933509 | NA |
| *Cytospora kantschavelii* | CXY 1383 | *Populus maximowiczii* | Jilin, China | KM034867 | NA | NA | NA | NA |
| *Cytospora kantschavelii* | CXY 1386 | *Populus maximowiczii* | Chongqing, China | KM034867 | NA | NA | NA | NA |
| ***Cytospora koelreutericola*** | **CFCC 56961^T^** | ***Koelreuteria paniculata*** | **Beijing, China** | **ON376918** | **ON390905** | **ON390908** | **ON390914** | **ON390923** |
| ***Cytospora koelreutericola*** | **CFCC 56970** | ***Koelreuteria paniculata*** | **Beijing, China** | **ON376917** | **ON390904** | **ON390907** | **ON390913** | **ON390922** |
| ***Cytospora koelreutericola*** | **CFCC 56971** | ***Koelreuteria paniculata*** | **Beijing, China** | **ON376919** | **ON390906** | **NA** | **ON390915** | **NA** |
| *Cytospora kuanchengensis* | CFCC 52464^T^ | *Castanea mollissima* | China | MK432616 | MK442940 | MK578076 | NA | NA |
| *Cytospora kuanchengensis* | CFCC 52465 | *Castanea mollissima* | China | MK432617 | MK442941 | MK578077 | NA | NA |
| *Cytospora longispora* | CBS 144236^T^ | *Prunus domestica* | California, USA | MG971905 | MG972054 | NA | MG971615 | MG971764 |
| *Cytospora longistiolata* | MFLUCC 16-0628 | *Salix* × *fragilis* | Russia | KY417734 | KY417700 | KY417802 | NA | NA |
| *Cytospora leucosperma* | CFCC 89622 | *Pyrus bretschneideri* | Gansu, China | KR045616 | KU710988 | KU710944 | KU710911 | KR045657 |
| *Cytospora leucosperma* | CFCC 89894 | *Pyrus bretschneideri* | Qinghai, China | KR045617 | KU710989 | KU710945 | KU710912 | KR045658 |
| *Cytospora leucostoma* | CFCC 50023 | *Cornus alba* | Shanxi, China | KR045635 | KU711003 | KU710964 | KU710926 | KR045676 |
| *Cytospora leucostoma* | CFCC 50024 | *Prunus pseudocerasus* | Qinghai, China | MH933640 | MH933547 | MH933605 | NA | MH933576 |
| *Cytospora leucostoma* | CFCC 53140 | *Prunus sibirica* | Beijing, China | MN854445 | MN850760 | MN850746 | MN850753 | MN861115 |
| *Cytospora leucostoma* | CFCC 53141 | *Prunus sibirica* | Beijing, China | MN854446 | MN850761 | MN850747 | MN850754 | MN861116 |
| *Cytospora leucostoma* | CFCC 53156 | *Juglans mandshurica* | Beijing, China | MN854447 | MN850762 | MN850748 | MN850755 | MN861117 |
| *Cytospora leucostoma* | CFCC 53167 | *Prunus armeniaca* | Xinjiang, China | MK673056 | MK673026 | MK672998 | MK672946 | MK672972 |
| *Cytospora leucostoma* | CFCC 53169 | *Prunus persica* | Beijing, China | MK673080 | MK673050 | MK673020 | MK672966 | MK672996 |
| *Cytospora leucostoma* | CFCC 53170 | *Prunus persica* | Beijing, China | MK673081 | MK673051 | MK673021 | MK672967 | MK672997 |
| *Cytospora leucostoma* | CFCC 54680 | *Corylus heterophylla* | Beijing, China | MW839857 | MW815941 | MW815955 | MW815890 | MW883973 |
| *Cytospora leucostoma* | CFCC 54681 | *Corylus heterophylla* | Beijing, China | MW839857 | MW815942 | MW815956 | MW815891 | MW883974 |
| *Cytospora leucostoma* | CFCC 54682 | *Corylus heterophylla* | Beijing, China | MW839857 | MW815943 | MW815957 | MW815892 | MW883975 |
| *Cytospora leucostoma* | CFCC 54683 | *Corylus heterophylla* | Beijing, China | MW839857 | MW815944 | MW815958 | MW815893 | MW883976 |
| *Cytospora lumnitzericola* | MFLUCC 17-0508^T^ | *Lumnitzera racernosa* | Tailand | MG975778 | MH253457 | MH253453 | NA | NA |
| *Cytospora mali* | CFCC 50028 | *Malus pumila* | Gansu, China | MH933641 | MH933548 | MH933606 | MH933513 | MH933577 |
| *Cytospora mali* | CFCC 50029 | *Malus pumila* | Ningxia, China | MH933642 | MH933549 | MH933607 | MH933514 | MH933578 |
| *Cytospora mali* | CFCC 50030 | *Malus pumila* | Shaanxi, China | MH933643 | MH933550 | MH933608 | MH933524 | MH933579 |
| *Cytospora mali* | CFCC 50031 | *Crataegus* sp. | Shanxi, China | KR045636 | KU711004 | KU710965 | KU710927 | KR045677 |
| *Cytospora mali* | CFCC 50044 | *Malus baccata* | Qinghai, China | KR045637 | KU711005 | KU710966 | KU710928 | KR045678 |
| *Cytospora mali-spectabilis* | CFCC 53181^T^ | *Malus spectabilis* ‘Royalty’ | Xinjiang, China | MK673066 | MK673036 | MK673006 | MK672953 | MK672982 |
| *Cytospora melnikii* | CFCC 89984 | *Rhus typhina* | Xinjiang, China | MH933678 | MH933551 | MH933609 | MH933515 | MH933580 |
| *Cytospora melnikii* | MFLUCC 15-0851 | *Malus domestica* | Russia | KY417735 | KY417701 | KY417803 | NA | NA |
| *Cytospora melnikii* | MFLUCC 16-0635 | *Populus nigra* var. *italica* | Russia | KY417736 | KY417702 | KY417804 | NA | NA |
| *Cytospora myrtagena* | CFCC 52454 | *Castanea mollissima* | China | MK432614 | MK442938 | MK578074 | NA | NA |
| *Cytospora myrtagena* | CFCC 52455 | *Castanea mollissima* | China | MK432615 | MK442939 | MK578075 | NA | NA |
| *Cytospora nivea* | MFLUCC 15-0860 | *Salix acutifolia* | Russia | KY417737 | KY417703 | KY417805 | NA | NA |
| *Cytospora nivea* | CFCC 89641 | *Elaeagnus angustifolia* | Ningxia, China | KF765683 | KU711006 | KU710967 | KU710929 | KR045679 |
| *Cytospora nivea* | CFCC 89643 | *Salix psammophila* | Shaanxi, China | KF765685 | NA | KU710968 | KP310863 | KP310829 |
| *Cytospora notastroma* | NE_TFR5 | *Populus tremuloides* | USA | JX438632 | NA | NA | JX438543 | NA |
| *Cytospora notastroma* | NE_TFR8 | *Populus tremuloides* | USA | JX438633 | NA | NA | JX438542 | NA |
| *Cytospora ochracea* | CFCC 53164^T^ | *Cotoneaster* sp. | Xinjiang, China | MK673060 | MK673030 | MK673001 | MK672949 | MK672976 |
| *Cytospora oleicola* | CBS 144248^T^ | *Olea europaea* | California, USA | MG971944 | MG972098 | NA | MG971660 | MG971752 |
| *Cytospora olivacea* | CFCC 53174 | *Prunus cerasifera* | Xinjiang, China | MK673058 | MK673028 | MK672999 | NA | MK672974 |
| *Cytospora olivacea* | CFCC 53175 | *Prunus dulcis* | Xinjiang, China | MK673062 | MK673032 | MK673003 | NA | MK672978 |
| *Cytospora olivacea* | CFCC 53176^T^ | *Sorbus tianschanica* | Xinjiang, China | MK673068 | MK673038 | MK673008 | MK672955 | MK672984 |
| *Cytospora olivacea* | CFCC 53177 | *Prunus virginiana* | Xinjiang, China | MK673071 | MK673041 | MK673011 | NA | MK672987 |
| *Cytospora palm* | CXY 1276 | *Cotinus coggygria* | Beijing, China | JN402990 | NA | NA | KJ781296 | NA |
| *Cytospora palm* | CXY 1280^T^ | *Cotinus coggygria* | Beijing, China | JN411939 | NA | NA | KJ781297 | NA |
| *Cytospora parakantschavelii* | MFLUCC 15-0857^T^ | *Populus* × *sibirica* | Russia | KY417738 | KY417704 | KY417806 | NA | NA |
| *Cytospora parakantschavelii* | MFLUCC 16-0575 | *Pyrus pyraster* | Russia | KY417739 | KY417705 | KY417807 | NA | NA |
| *Cytospora parapistaciae* | CBS 144506^T^ | *Pistacia vera* | California, USA | MG971804 | MG971954 | NA | MG971519 | MG971669 |
| *Cytospora parasitica* | MFLUCC 15-0507^T^ | *Malus domestica* | Russia | KY417740 | KY417706 | KY417808 | NA | NA |
| *Cytospora parasitica* | XJAU 2542-1 | *Malus* sp. | Xinjiang, China | MH798884 | NA | NA | MH813452 | NA |
| *Cytospora parasitica* | CFCC 53171 | *Malus pumila* | Xinjiang, China | MK673061 | MK673031 | MK673002 | MK672950 | MK672977 |
| *Cytospora parasitica* | CFCC 53172 | *Malus pumila* | Xinjiang, China | MK673069 | MK673039 | MK673009 | MK672956 | MK672985 |
| *Cytospora parasitica* | CFCC 53173 | *Berberis* sp. | Xinjiang, China | MK673070 | MK673040 | MK673010 | MK672957 | MK672986 |
| *Cytospora paratranslucens* | MFLUCC 15-0506^T^ | *Populus alba* var. *bolleana* | Russia | KY417741 | KY417707 | KY417809 | NA | NA |
| *Cytospora paratranslucens* | MFLUCC 16-0627 | *Populus alba* | Russia | KY417742 | KY417708 | KY417810 | NA | NA |
| *Cytospora phialidica* | MFLUCC 17-2498 | *Alnus glutinosa* | Italy | MT177932 | NA | MT432209 | MT454014 | NA |
| *Cytospora piceae* | CFCC 52841^T*^ | *Picea crassifolia* | Xinjiang, China | MH820398 | MH820406 | MH820395 | MH820402 | MH820387 |
| *Cytospora piceae* | CFCC 52842* | *Picea crassifolia* | Xinjiang, China | MH820399 | MH820407 | MH820396 | MH820403 | MH820388 |
| *Cytospora pingbianensis* | MFLUCC 18-1204^T^ | Undefined wood | Yunnan, China | MK912135 | MN685817 | MN685826 | NA | NA |
| *Cytospora pistaciae* | CBS 144238^T^ | *Pistacia vera* | California, USA | MG971802 | MG971952 | NA | MG971517 | MG971667 |
| *Cytospora platanicola* | MFLU 17-0327 | *Platanus hybrida* | Italy | MH253451 | MH253449 | MH253450 | NA | NA |
| *Cytospora platycladi* | CFCC 50504^T^ | *Platycladus orientalis* | Yunnan, China | MH933645 | MH933552 | MH933610 | MH933516 | MH933581 |
| *Cytospora platycladi* | CFCC 50505 | *Platycladus orientalis* | Yunnan, China | MH933646 | MH933553 | MH933611 | MH933517 | MH933582 |
| *Cytospora platycladi* | CFCC 50506 | *Platycladus orientalis* | Yunnan, China | MH933647 | MH933554 | MH933612 | MH933518 | MH933583 |
| *Cytospora platycladicola* | CFCC 50038^T^ | *Platycladus orientalis* | Gansu, China | KT222840 | MH933555 | MH933613 | MH933519 | MH933584 |
| *Cytospora platycladicola* | CFCC 50039 | *Platycladus orientalis* | Gansu, China | KR045642 | KU711008 | KU710973 | KU710931 | KR045683 |
| *Cytospora plurivora* | CBS 144239^T^ | *Olea europaea* | California, USA | MG971861 | MG972010 | NA | MG971572 | MG971726 |
| *Cytospora populicola* | CBS 144240 | *Populus deltoides* | California, USA | MG971891 | MG972040 | NA | MG971601 | MG971757 |
| *Cytospora populina* | CFCC 89644^T^ | *Salix psammophila* | Shaanxi, China | KF765686 | KU711007 | KU710969 | KU710930 | KR045681 |
| *Cytospora populinopsis* | CFCC 50032^T^ | *Sorbus aucuparia* | Ningxia, China | MH933648 | MH933556 | MH933614 | MH933520 | MH933585 |
| *Cytospora populinopsis* | CFCC 50033 | *Sorbus aucuparia* | Ningxia, China | MH933649 | MH933557 | MH933615 | MH933521 | MH933586 |
| *Cytospora predappioensis* | MFLUCC 17-2458^T^ | *Platanus hybrida* | Italy | MG873484 | NA | NA | NA | NA |
| *Cytospora prunicola* | MFLU 17-0995^T^ | *Prunus* sp. | Italy | MG742350 | MG742353 | MG742352 | NA | NA |
| *Cytospora pruni-mume* | CFCC 53179 | *Prunus armeniaca* | Xinjiang, China | MK673057 | MK673027 | NA | MK672947 | MK672973 |
| *Cytospora pruni-mume* | CFCC 53180^T^ | *Prunus mume* | Xinjiang, China | MK673067 | MK673037 | MK673007 | MK672954 | MK672983 |
| *Cytospora pruinopsis* | CFCC 50034^T^ | *Ulmus pumila* | Shaanxi, China | KP281259 | KP310836 | KU710970 | KP310849 | KP310819 |
| *Cytospora pruinopsis* | CFCC 50035 | *Ulmus pumila* | Jilin, China | KP281260 | KP310837 | KU710971 | KP310850 | KP310820 |
| *Cytospora pruinopsis* | CFCC 53153 | *Ulmus pumila* | Beijing, China | MN854451 | MN850763 | MN850752 | MN850759 | MN861121 |
| *Cytospora pruinosa* | CFCC 50036 | *Syringa oblata* | Qinghai, China | KP310800 | KP310832 | NA | KP310845 | KP310815 |
| *Cytospora pruinosa* | CFCC 50037 | *Syringa oblata* | Qinghai, China | MH933650 | MH933558 | NA | MH933522 | MH933589 |
| *Cytospora pubescentis* | MFLUCC 18-1201^T^ | *Quercus pubescens* | Forlì-Cesena, Italy | MK912130 | MN685812 | MN685821 | NA | NA |
| *Cytospora punicae* | CBS 144244 | *Punica granatum* | California, USA | MG971943 | MG972091 | NA | MG971654 | MG971798 |
| *Cytospora quercicola* | MFLU 17-0881 | *Quercus* sp. | Italy | MF190128 | NA | NA | NA | NA |
| *Cytospora quercicola* | MFLUCC 14-0867^T^ | *Quercus* sp. | Italy | MF190129 | NA | NA | NA | NA |
| *Cytospora ribis* | CFCC 50026 | *Ulmus pumila* | Qinghai, China | KP281267 | KP310843 | KU710972 | KP310856 | KP310826 |
| *Cytospora ribis* | CFCC 50027 | *Ulmus pumila* | Qinghai, China | KP281268 | KP310844 | NA | KP310857 | KP310827 |
| *Cytospora rosae* | MFLU 17-0885 | *Rosa canina* | Italy | MF190131 | NA | NA | NA | NA |
| *Cytospora rosicola* | CF 20197024^T^ | *Rosa* sp. | Tibet, China | MK673079 | MK673049 | MK673019 | MK672965 | MK672995 |
| *Cytospora rosigena* | MFLUCC 18-0921^T^ | *Rosa* sp. | Russia | MN879872 | NA | NA | NA | NA |
| *Cytospora rostrata* | CFCC 89909^T^ | *Salix cupularis* | Gansu, China | KR045643 | KU711009 | KU710974 | KU710932 | KR045684 |
| *Cytospora rostrata* | CFCC 89910 | *Salix cupularis* | Gansu, China | KR045644 | KU711010 | KU710975 | KU710933 | NA |
| *Cytospora rusanovii* | MFLUCC 15-0853 | *Populus* × *sibirica* | Russia | KY417743 | KY417709 | KY417811 | NA | NA |
| *Cytospora rusanovii* | MFLUCC 15-0854^T^ | *Salix babylonica* | Russia | KY417744 | KY417710 | KY417812 | NA | NA |
| *Cytospora salicacearum* | MFLUCC 15-0509 | *Salix alba* | Russia | KY417746 | KY417712 | KY417814 | NA | NA |
| *Cytospora salicacearum* | MFLUCC 15-0861 | *Salix* × *fragilis* | Russia | KY417745 | KY417711 | KY417813 | NA | NA |
| *Cytospora salicacearum* | MFLUCC 16-0587 | *Prunus cerasus* | Russia | KY417742 | KY417708 | KY417810 | NA | NA |
| *Cytospora salicacearum* | MFLUCC 16-0576 | *Populus nigra* var. *italica* | Russia | KY417741 | KY417707 | KY417809 | NA | NA |
| *Cytospora salicicola* | MFLUCC 14-1052^T^ | *Salix alba* | Russia | KU982636 | KU982637 | NA | NA | NA |
| *Cytospora salicicola* | MFLUCC 15-0866 | *Salix* sp. | Thailand | KY417749 | KY417715 | KY417817 | NA | NA |
| *Cytospora salicina* | MFLUCC 15-0862 | *Salix alba* | Russia | KY417750 | KY417716 | KY417818 | NA | NA |
| *Cytospora salicina* | MFLUCC 16-0637 | *Salix* × *fragilis* | Russia | KY417751 | KY417717 | KY417819 | NA | NA |
| *Cytospora schulzeri* | CFCC 50040 | *Malus domestica* | Ningxia, China | KR045649 | KU711013 | KU710980 | KU710936 | KR045690 |
| *Cytospora schulzeri* | CFCC 50042 | *Malus pumila* | Gansu, China | KR045650 | KU711014 | KU710981 | KU710937 | KR045691 |
| *Cytospora sibiraeae* | CFCC 50045^T^ | *Sibiraea angustata* | Gansu, China | KR045651 | KU711015 | KU710982 | KU710938 | KR045692 |
| *Cytospora sibiraeae* | CFCC 50046 | *Sibiraea angustata* | Gansu, China | KR045652 | KU711015 | KU710983 | KU710939 | KR045693 |
| *Cytospora sophorae* | CFCC 50047 | *Styphnolobium japonicum* | Shanxi, China | KR045653 | KU711017 | KU710984 | KU710940 | KR045694 |
| *Cytospora sophorae* | CFCC 50048 | *Magnolia grandiflora* | Shanxi, China | MH820401 | MH820409 | MH820397 | MH820405 | MH820390 |
| *Cytospora sophorae* | CFCC 89598 | *Styphnolobium japonicum* | Gansu, China | KR045654 | KU711018 | KU710985 | KU710941 | KR045695 |
| *Cytospora sophoricola* | CFCC 89596 | *Styphnolobium japonicum*  var. *pendula* | Gansu, China | KR045656 | KU711020 | KU710987 | KU710943 | KR045697 |
| *Cytospora sophoricola* | CFCC 89595^T^ | *Styphnolobium japonicum*  var. *pendula* | Gansu, China | KR045655 | KU711019 | KU710986 | KU710942 | KR045696 |
| *Cytospora sophoriopsis* | CFCC 89600^T^ | *Styphnolobium japonicum* | Gansu, China | KR045623 | KU710992 | KU710951 | KU710915 | KP310817 |
| *Cytospora sorbi* | MFLUCC 16-0631^T^ | *Sorbus aucuparia* | Russia | KY417752 | KY417718 | KY417820 | NA | NA |
| *Cytospora sorbicola* | MFLUCC 16-0584^T^ | *Acer pseudoplatanus* | Russia | KY417755 | KY417721 | KY417823 | NA | NA |
| *Cytospora sorbicola* | MFLUCC 16-0633 | *Cotoneaster melanocarpus* | Russia | KY417758 | KY417724 | KY417826 | NA | NA |
| *Cytospora sorbina* | CF 20197660^T^ | *Sorbus tianschanica* | Xinjiang, China | MK673052 | MK673022 | NA | MK672943 | MK672968 |
| *Cytospora spiraeae* | CFCC 50049^T^ | *Spiraea salicifolia* | Gansu, China | MG707859 | MG708196 | MG708199 | NA | NA |
| *Cytospora spiraeae* | CFCC 50050 | *Spiraea salicifolia* | Gansu, China | MG707860 | MG708197 | MG708200 | NA | NA |
| *Cytospora spiraeicola* | CFCC 53138^T^ | *Spiraea salicifolia* | Beijing, China | MN854448 | NA | MN850749 | MN850756 | MN861118 |
| *Cytospora spiraeicola* | CFCC 53139 | *Tilia nobilis* | Beijing, China | MN854449 | NA | MN850750 | MN850757 | MN861119 |
| *Cytospora tamaricicola* | CFCC 50507 | *Rosa multifolora* | Yunnan, China | MH933651 | MH933559 | MH933616 | MH933525 | MH933587 |
| *Cytospora tamaricicola* | CFCC 50508^T^ | *Tamarix chinensis* | Yunnan, China | MH933652 | MH933560 | MH933617 | MH933523 | MH933588 |
| *Cytospora tanaitica* | MFLUCC 14-1057^T^ | *Betula pubescens* | Russia | KT459411 | KT459413 | NA | NA | NA |
| *Cytospora thailandica* | MFLUCC 17-0262^T^ | *Xylocarpus moluccensis* | Thailand | MG975776 | MH253459 | MH253455 | NA | NA |
| *Cytospora thailandica* | MFLUCC 17-0263^T^ | *Xylocarpus moluccensis* | Thailand | MG975777 | MH253460 | MH253456 | NA | NA |
| *Cytospora tibetensis* | CF 20197026 | *Cotoneaster* sp. | Tibet, China | MK673076 | MK673046 | MK673016 | MK672962 | MK672992 |
| *Cytospora tibetensis* | CF 20197029 | *Cotoneaster* sp. | Tibet, China | MK673077 | MK673047 | MK673017 | MK672963 | MK672993 |
| *Cytospora tibetensis* | CF 20197032^T^ | *Cotoneaster* sp. | Tibet, China | MK673078 | MK673048 | MK673018 | MK672964 | MK672994 |
| *Cytospora tibouchinae* | CPC 26333^T^ | *Tibouchina semidecandra* | France | KX228284 | NA | NA | NA | NA |
| *Cytospora translucens* | CXY 1351 | *Populus davidiana* | China | KM034874 | NA | NA | NA | KM034895 |
| *Cytospora translucens* | CXY 1359 | *Populus* × Beijingensis | Beijing, China | KM034871 | NA | NA | NA | KM034894 |
| *Cytospora ulmi* | MFLUCC 15-0863^T^ | *Ulmus minor* | Russia | KY417759 | NA | NA | NA | NA |
| *Cytospora verrucosa* | CFCC 53157 ^T^ | *Platycladus orientalis* | Beijing, China | MW418408 | NA | MW422911 | MW422923 | MW422935 |
| *Cytospora verrucosa* | CFCC 53158 | *Platycladus orientalis* | Beijing, China | MW418410 | MW422901 | MW422913 | MW422925 | MW422937 |
| *Cytospora verrucosa* | CFCC 54369 | *Platycladus orientalis* | Beijing, China | MW418409 | NA | MW422912 | MW422924 | MW422936 |
| *Cytospora verrucosa* | CFCC 54370 | *Platycladus orientalis* | Beijing, China | MW418411 | MW422902 | MW422914 | MW422926 | MW422938 |
| *Cytospora vinacea* | CBS 141585^T^ | *Vitis interspecific*  hybrid ‘Vidal’ | USA | KX256256 | NA | NA | KX256277 | KX256235 |
| *Cytospora viridistroma* | CBS 202.36^T^ | *Cercis canadensis* Castigl. | USA | MN172408 | NA | NA | MN271853 | NA |
| *Cytospora viticola* | Cyt2 | *Vitis interspecific*  hybrid ‘Frontenac’ | USA | KX256238 | NA | NA | KX256259 | KX256217 |
| *Cytospora viticola* | CBS 141586^T^ | *Vitis vinifera*  ‘CabernetFranc’ | USA | KX256239 | NA | NA | KX256260 | KX256218 |
| *Cytospora xinjiangensis* | CFCC 53182 | *Rosa* sp. | Xinjiang, China | MK673064 | MK673034 | MK673004 | MK672951 | MK672980 |
| *Cytospora xinjiangensis* | CFCC 53183^T^ | *Rosa* sp. | Xinjiang, China | MK673065 | MK673035 | MK673005 | MK672952 | MK672981 |
| *Cytospora xinglongensis* | CFCC 52458^T^ | *Castanea mollissima* | China | MK432622 | MK442946 | MK578082 | NA | NA |
| *Cytospora xinglongensis* | CFCC 52459 | *Castanea mollissima* | China | MK432623 | MK442947 | MK578083 | NA | NA |
| *Cytospora xylocarpi* | MFLUCC 17-0251^T^ | *Xylocarpus granatum* | Thailand | MG975775 | MH253458 | MH253454 | NA | NA |
| *Diaporthe vaccinii* | CBS 160.32 | *Vaccinium macrocarpon* | USA | KC343228 | JQ807297 | NA | KC343954 | KC344196 |

^1^ Acronyms: ATCC: American Type Culture Collecton, Virginia, USA; BBH: BIOTEC Bangkok Herbarium, National Science and Technology Development Agency, Thailand; CBS: Westerdijk Fungal Biodiversity Institute (CBS-KNAW Fungal Biodiversity Centre), Utrecht, The Netherlands; CFCC: China Forestry Culture Collection Centre, Beijing, China; CMW: Culture collection of Michael Wingfield, University of Pretoria, South Africa; CPC: Culture collection of Pedro Crous, The Netherlands; IMI: Culture collection of the International Mycological Institute, CABI Bioscience, Egham, Surrey, UK; MFLU: Mae Fah Luang University herbarium, Thailand; MFLUCC: Mae Fah Luang University Culture Collection, Thailand; MUCC: Murdoch University Culture Collection, Perth, Australia; NE: Gerard Adams collections, University of Nebraska, Lincoln NE, USA; PPRI: Culture collection of the Plant Protection Research Institute, Agriculture Research Center, Pretoria, South Africa; XJAU: Xinjiang Agricultural University, Xinjiang, China; NA: not applicable. All the new isolates used in this study are in bold and the type materials are marked with T.

**Table S5.** Strains of *Diatrypaceae* used in the molecular analyses in this study.

| **Species** | **Strain^1^** | **Host** | **Origin** | **GenBank accession numbers** | |
| --- | --- | --- | --- | --- | --- |
|  |  |  |  | **ITS** | **TUB2** |
| ***Allocryptovalsa castanea*** | **CFCC 52428** | *Castanea mollissima* | China | MW632945 | MW656393 |
| *Allocryptovalsa castanea* | CFCC 52427 | *Juglans regia* | China | MW632944 | MW656392 |
| *Allocryptovalsa castanea* | CFCC 52429 | *Castanea mollissima* | China | MW632946 | MW656394 |
| ***Allocryptovalsa castaneicola*** | **CFCC 52432** | *Castanea mollissima* | China | MW632947 | MW656395 |
| *Allocryptovalsa castaneicola* | CFCC 56962* | *Koelreuteria paniculata* | Beijing, China | ON376923 | ON390929 |
| *Allocryptovalsa castaneicola* | CFCC 56963* | *Koelreuteria paniculata* | Beijing, China | ON376922 | ON390928 |
| *Allocryptovalsa castaneicola* | CFCC 56969* | *Koelreuteria paniculata* | Beijing, China | ON376924 | ON390930 |
| ***Allocryptovalsa cryptovalsoidea*** | **HVFIG02** | *Ficus carica* | Australia | HQ692573 | HQ692524 |
| *Allocryptovalsa cryptovalsoidea* | HVFIG05 | *Ficus carica* | Australia | HQ692574 | HQ692525 |
| ***Allocryptovalsa elaeidis*** | **MFLUCC 15-0707** | *Elaeis guineensis* | Thailand | MN308410 | MN340296 |
| ***Allocryptovalsa polyspora*^TS^** | **MFLUCC 17-0364** | *Hevea brasiliensis* | Thailand | MF959500 | NA |
| *Allocryptovalsa rabenhorstii* | WA07CO | *Vitis vinifera* | Australia | HQ692620 | HQ692522 |
| *Allocryptovalsa rabenhorstii* | WA08CB | *Vitis vinifera* | Australia | HQ692619 | HQ692523 |
| ***Allocryptovalsa truncata*** | **NFCCI-4520** | NA | Inidia | MK990279 | NA |
| ***Allodiatrype arengae*^TS^** | **MFLUCC 15-0713** | *Arenga pinnata* | Thailand | MN308411 | MN340297 |
| ***Allodiatrype elaeidicola*** | **MFLUCC 15-0737a** | *Elaeis guineensis* | Thailand | MN308415 | MN340299 |
| ***Allodiatrype elaeidicola*** | **MFLUCC 15-0737b** | *Elaeis guineensis* | Thailand | MN308416 | NA |
| ***Allodiatrype elaeidis*** | **MFLUCC 15-0708a** | *Elaeis guineensis* | Thailand | MN308412 | MN340298 |
| ***Allodiatrype elaeidis*** | **MFLUCC 15-0708b** | *Elaeis guineensis* | Thailand | MN308413 | NA |
| *Allodiatrype thailandica* | MFLUCC 15-0711 | *Calamus* sp. | Thailand | MN308414 | NA |
| ***Allodiatrype thailandica*** | **MFLUCC 14-1210** | NA | Thailand | KU315392 | NA |
| *Anthostoma decipiens*^TS^ | IPV-FW349 | NA | Italy | AM399021 | NA |
| *Anthostoma decipiens*^TS^ | JL567 | *Vitis vinifera* | Spain | JN975370 | JN975407 |
| *Cryptosphaeria eunomia* var. *eunomia* | CBS 216.87 | *Fraxinus excelsior* | Switzerland | AJ302417 | NA |
| *Cryptosphaeria eunomia* var. *fraxini* | CBS 223.87 | *Fraxinus excelsior* | Switzerland | AJ302421 | KT425166 |
| ***Cryptosphaeria ligniota*** | **CBS 273.87** | *Populus tremula* | Switzerland | KT425233 | KT425168 |
| ***Cryptosphaeria multicontinentalis*** | **CBS 132918** | *Populus balsamifera* subsp. *trichocarpa* | Australia | KT425237 | KT425172 |
| ***Cryptosphaeria pullmanensis*** | **ATCC 52655** | *Populus trichocarpa* | USA | KT425235 | KT425170 |
| ***Cryptosphaeria subcutanea*** | **CBS 240.87** | NA | Norway | KT425232 | KT425167 |
| *Cryptosphaeria subcutanea* | DSUB100A | NA | Norway | KT425189 | KT425124 |
| *Cryptovalsa ampelina* | A001 | NA | Australia | GQ293901 | GQ293972 |
| *Cryptovalsa ampelina* | DRO101 | NA | USA | GQ293902 | GQ293982 |
| ***Diatrypasimilis australiensis*^TS^** | **ATCC MYA-3540** | *Rhizophora* sp. | Australia | FJ430590 | NA |
| ***Diatrype betulae*** | **CFCC 52416** | *Betula davurica* | China | MW632943 | MW656391 |
| ***Diatrype brunneospora*** | **CNP01** | *Acacia longifolia* subsp. *sophorae* | Australia | HM581946 | HQ692478 |
| *Diatrype bullata* | UCDDCh400 | NA | USA | DQ006946 | DQ007002 |
| ***Diatrype castaneicola*** | **CFCC 52425** | *Castanea mollissima* | China | MW632941 | MW656389 |
| *Diatrype castaneicola* | CFCC 52426 | *Castanea mollissima* | China | MW632942 | MW656390 |
| *Diatrype disciformis*^TS^ | CBS 205.87 | *Fagus sylvatica* | Switzerland | AJ302437 | NA |
| *Diatrype disciformis*^TS^ | GB 5815 | *Fagus grandifolia* | USA | AJ302423 | NA |
| ***Diatrype iranensis*** | **IRAN 2280C** | *Quercus brantii* | Iran | KM245033 | NA |
| ***Diatrype macrospora*** | **IRAN 2344C** | *Quercus brantii* | Iran | KR605648 | NA |
| ***Diatrype palmicola*** | **MFLUCC 11-0018** | *Caryota urens* | Thailand | KP744438 | NA |
| *Diatrype palmicola* | MFLUCC 11-0020 | *Caryota urens* | Thailand | KP744439 | NA |
| ***Diatrype quercicola*** | **CFCC 52418** | *Quercus mongolica* | China | MW632938 | MW656386 |
| *Diatrype quercicola* | CFCC 52419 | *Quercus mongolica* | China | MW632939 | MW656387 |
| *Diatrype quercicola* | CFCC 52420 | *Quercus mongolica* | China | MW632940 | MW656388 |
| *Diatrype spilomea* | CBS 212.87 | *Acer campestre* | Switzerland | AJ302433 | NA |
| *Diatrype stigma* | DCASH200 | *Quercus* sp. | USA | GQ293947 | GQ294003 |
| *Diatrype stigma* | UCDDCash200 | NA | NA | DQ006945 | DQ007003 |
| *Diatrype undulata* | CBS 271.87 | *Betula* sp. | Switzerland | AJ302436 | NA |
| *Diatrype virescens* | CBS 128344 | NA | NA | MH864890 | NA |
| *Diatrypella atlantica* | HUEFS 136873 | unidentified plant | Brazil | KM396614 | KR259647 |
| ***Diatrypella atlantica*** | **HUEFS 194228** | unidentified plant | Brazil | KM396615 | KR363998 |
| *Diatrypella banksiae* | CPC 29054 | *Banksia coccinia* | Australia | KY173401 | NA |
| ***Diatrypella banksiae*** | **CPC 29118** | *Banksia formosa* | Australia | KY173402 | NA |
| ***Diatrypella betulae*** | **CFCC 52406** | *Betula albosinensis* | China | MW632931 | MW656379 |
| *Diatrypella betulae* | CFCC 52404 | *Betula albosinensis* | China | MW632929 | MW656377 |
| *Diatrypella betulae* | CFCC 52405 | *Betula albosinensis* | China | MW632930 | MW656378 |
| ***Diatrypella betulicola*** | **CFCC 52411** | *Betula davurica* | China | MW632935 | MW656383 |
| *Diatrypella betulicola* | CFCC 52412 | *Betula platyphylla* | China | MW632936 | MW656384 |
| *Diatrypella delonicis* | MFLU 16-1032 | *Delonix regia* | Thailand | MH812995 | MH847791 |
| *Diatrypella delonicis* | MFLUCC 15-1014 | *Delonix regia* | Thailand | MH812994 | MH847790 |
| ***Diatrypella elaeidis*** | **MFLUCC 15-0279** | *Elaeis guineensis* | Thailand | MN308417 | MN340300 |
| *Diatrypella favacea*^TSQ^ | CBS 198.49 | *Betula pendula* | NA | MH856491 | NA |
| *Diatrypella favacea*^TSQ^ | CFCC 52409 | *Betula platyphylla* | China | MW632934 | MW656382 |
| *Diatrypella favacea*^TSQ^ | DL26C | *Betula* sp. | Netherlands | AJ302440 | NA |
| *Diatrypella favacea*^TSQ^ | R191 | *Betula* sp. | Lithuania | JN689955 | NA |
| *Diatrypella frostii* | UFMGCB 1917 | *Solanum cernuum* | Brazil | HQ377280 | NA |
| ***Diatrypella heveae*** | **MFLUCC 17-0368** | *Hevea brasiliensis* | Thailand | MF959501 | MG334557 |
| ***Diatrypella hubeiensis*** | **CFCC 52413** | *Betula davurica* | China | MW632937 | MW656385 |
| *Diatrypella major* | ANM 1947 | NA | USA | KU320613 | NA |
| *Diatrypella prominens* | ATCC 64182 | *Plantanus* sp. | USA | AJ302442 | NA |
| *Diatrypella pulvinata* | DL29C | NA | NA | AJ302443 | NA |
| *Diatrypella pulvinata* | H048 | *Salix alba* | Czech Republic | FR715523 | FR715495 |
| *Diatrype quercina* | F-091966 | *Quercus faginea* | Spain | AJ302444 | NA |
| *Diatrypella shennongensis* | CFCC 52414 | *Betula albosinensis* | China | MW632932 | MW656380 |
| ***Diatrypella shennongensis*** | **CFCC 52415** | *Betula albosinensis* | China | MW632933 | MW656381 |
| ***Diatrypella tectonae*** | **MFLUCC 12-0172a** | *Tectona grandis* | Thailand | KY283084 | NA |
| ***Diatrypella tectonae*** | **MFLUCC 12-0172b** | *Tectona grandis* | Thailand | KY283085 | NA |
| *Diatrypella verruciformis*^TSQ^ | UCROK1467 | *Quercus agrifolia* | USA | JX144793 | JX174093 |
| *Diatrypella verruciformis*^TSQ^ | UCROK754 | *Quercus agrifolia* | USA | JX144783 | JX174083 |
| *Diatrypella vulgaris* | HVFRA02 | *Fraxinus angustifolia* | Australia | HQ692591 | HQ692503 |
| ***[Diatrypella vulgaris](https://www.ncbi.nlm.nih.gov/nuccore/MH876328.1)*** | **HVGRF03** | *Citrus paradisi* | Australia | HQ692590 | HQ692502 |
| ***Diatrypella yunnanensis*** | **JZBH3380001** | unidentified plant | China | MN653008 | MN887112 |
| *Eutypa astroidea* | CBS 292.87 | NA | Switzerland | AJ302458 | DQ006966 |
| *Eutypa consobrina* | STEU 8153 | NA | South Africa | MF359628 | MF359661 |
| ***Eutypa cremea*** | **STEU 8082** | *Vitis vinifera* | South Africa | KY111656 | KY111598 |
| *Eutypa cremea* | STEU 8410 | *Prunus armeniaca* | South Africa | KY752765 | KY752789 |
| *Eutypa crustata* | CBS 210.87 | *Ulmus* sp. | France | AJ302448 | DQ006968 |
| *Eutypa flavovirens* | CBS 272.87 | *Quercus ilex* | France | AJ302457 | DQ006959 |
| ***Eutypa guttulata*** | **HUEFS 192075** | unidentified plant | Brazil | KM396637 | NA |
| *Eutypa laevata* | CBS 291.87 | *Salix* sp. | Switzerland | HM164737 | HM164771 |
| *Eutypa lata*^TS^ | EP18 | *Vitis vinifera* | New South Wales | HQ692611 | HQ692501 |
| *Eutypa lata*^TS^ | JL399 | Cabernet Sauvignon | Spain | JN975339 | JN975376 |
| *Eutypa lata*^TS^ | ATCC 28120 | NA | Australia | DQ006948 | DQ006975 |
| *Eutypa lata*^TS^ | CBS 622.84 | *Vitis vinifera* | Italy | AJ302446 | DQ006964 |
| *Eutypa lata* var. *aceris* | CBS 217.87 | *Acer campestre* | France | HM164734 | HM164768 |
| *Eutypa lejoplaca* | 020202-3 | *Acer pseudoplatanus* | Switzerland | AY684238 | AY684197 |
| *Eutypa lejoplaca* | 020202-5 | *Acer pseudoplatanus* | Switzerland | AY684221 | AY684196 |
| *Eutypa leptoplaca* | CBS 287.87 | *Frangula alnus* | Switzerland | AY684226 | AY684204 |
| *Eutypa leptoplaca* | CBS 288.87 | *Cyssus hypoglauca* | Australia | AY684227 | AY684205 |
| *Eutypa maura* | CBS 219.87 | *Vitis vinifera* | Switzerland | DQ006926 | DQ006967 |
| *Eutypa petrakii* var. *hederae* | CBS 285.87 | NA | Switzerland | MH862077 | NA |
| *Eutypa petrakii* var*. petrakii* | CBS 245.87 | NA | Norway | AJ302456 | DQ006971 |
| *Eutypa sparsa* | 3802-3a | *Populus* sp. | Switzerland | AY684219 | AY684200 |
| *Eutypa sparsa* | 3802-3b | *Populus* sp. | Switzerland | AY684220 | AY684201 |
| *Eutypa tetragona* | CBS 284.87 | *Sarothamnus scoparius* | France | DQ006923 | DQ006960 |
| ***Eutypella australiensis*** | **CNP03** | *Acacia longifolia* subsp. *sophorae* | Australia | HM581945 | HQ692479 |
| ***Eutypella cearensis*** | **HUEFS 131070** | Unidentified plant | Brazil | KM396639 | NA |
| *Eutypella cerviculata*^TS^ | CBS 221.87 | *Alnus glutinosa* | Switzerland | AJ302468 | NA |
| *Eutypella cerviculata*^TS^ | M68 | *Alnus glutinosa* | Latvia | JF340269 | NA |
| *Eutypella citricola* | HVGRF01 | *Citrus sinensis* | Australia | HQ692589 | HQ692521 |
| *Eutypella citricola* | HVVIT07 | *Vitis vinifera* | Australia | HQ692579 | HQ692512 |
| *Eutypella citricola* | CFCC 52433 | *Morus alba* | China | MW632948 | MW656396 |
| *Eutypella citricola* | CFCC 52434 | *Morus alba* | China | MW632949 | MW656397 |
| *Eutypella citricola* | CFCC 56968* | *Koelreuteria paniculata* | Beijing, China | ON376926 | ON390924 |
| *Eutypella citricola* | CFCC 56972* | *Koelreuteria paniculata* | Beijing, China | ON376925 | ON390925 |
| *Eutypella leprosa* | STEU 8189 | NA | South Africa | MF359637 | MF359672 |
| *Eutypella leprosa* | STEU 8190 | NA | South Africa | MF359638 | MF359673 |
| *Eutypella microtheca* | ADEL200 | *Ulmus procera* | Australia | HQ692559 | HQ692527 |
| *Eutypella microtheca* | BCMX01 | *Vitis vinifera* | Mexico | KC405563 | KC405560 |
| *Eutypella microtheca* | CBS 128337 | *Citrus paradisi* | Australia | MH864886 | NA |
| ***Eutypella persica*** | **IRAN 2540C** | *Alnus* sp. | Iran | KX828144 | NA |
| ***Eutypella quercina*** | **IRAN 2543C** | *Quercus* sp*.* | Iran | KX828139 | NA |
| ***Eutypella semicircularis*** | **MP4669** | *Alnus acuminata* | Panama | JQ517314 | NA |
| *Eutypella vitis* | UCD2291AR | *Vitis vinifera* | USA | HQ288224 | HQ288303 |
| *Eutypella vitis* | UCD2428TX | *Vitis vinifera* | Texas, USA | FJ790851 | GU294726 |
| ***Halocryptosphaeria bathurstensis*^TS^** | **NFCCI-4248** | *Avicennia marina* | India | MN061366 | MN431496 |
| ***Halocryptovalsa salicorniae*^TS^** | **MFLUCC 15-0185** | *Salicornia sp.* | Thailand | MH304410 | MH370274 |
| ***Halodiatrype avicenniae*^TS^** | **MFLUCC 15-0953** | *Avicennia* sp. | Thailand | KX573916 | KX573931 |
| ***Halodiatrype salinicola*^TS^** | **MFLUCC 15-1277** | submerged marine wood | Thailand | KX573915 | KX573932 |
| ***Monosporascus cannonballus*^TS^** | **ATCC 26931** | NA | NA | FJ430598 | NA |
| *Monosporascus cannonballus*^TS^ | CMM 3646 | *Boerhavia sp.* | Brazil | JX971617 | NA |
| ***Neoeutypella baoshanensis*^TS^** | **BAP101** | *Pinus armandii* | China | MH822887 | MH822888 |
| *Neoeutypella baoshanensis* ^TS^ | CBS 274.87 | *Ficus carica* | France | AJ302460 | NA |
| *Neoeutypella baoshanensis* ^TS^ | GL08362 | NA | China | JX241652 | NA |
| *Pedumispora rhizophorae*^TS^ | BCC44877 | *Rhizophora apiculata* | Thailand | KJ888853 | NA |
| *Pedumispora rhizophorae*^TS^ | BCC44878 | *Rhizophora apiculata* | Thailand | KJ888854 | NA |
| *Peroneutypa alsophila* | CBS 250.87 | *Arthrocnemum fruticosum* | France | AJ302467 | NA |
| *Peroneutypa curvispora* | HUEFS 136877 | unidentified plant | Brazil | KM396641 | NA |
| ***Peroneutypa diminutispora*** | **HUEFS 192196** | unidentified plant | Brazil | KM396647 | NA |
| ***Peroneutypa indica*** | **NFCCI-4393** | *Suaeda monoica* | India | MN061368 | MN431498 |
| *Peroneutypa kochiana* | F-092373 | *Atriplex halimus* | Spain | AJ302462 | NA |
| ***Peroneutypa longiasca*** | **MFLUCC 17-0371** | *Hevea brasiliensis* | Thailand | MF959502 | NA |
| ***Peroneutypa mackenziei*** | **MFLUCC 16-0072** | undetermined decaying wood | Thailand | KY283083 | NA |
| ***Peroneutypa microasca*** | **BAFC 51550** | unidentifed wood | Argentina | KF964566 | KF964572 |
| ***Peroneutypa polysporae*** | **NFCCI-4392** | *Suaeda monoica* | India | MN061367 | MN431497 |
| *Peroneutypa scoparia* | CBS 242.87 | *Robinia pseudoacacia* | France | AJ302465 | NA |
| *Peroneutypa scoparia* | CFCC 56957* | *Koelreuteria paniculata* | Beijing, China | ON376921 | ON390927 |
| *Peroneutypa scoparia* | CFCC 56964* | *Koelreuteria paniculata* | Beijing, China | ON376920 | ON390926 |
| *Quaternaria quaternata* | GNF13 | *Fagus* sp. | Iran | KR605645 | KY352464 |
| *Quaternaria quaternata* | CBS 278.87 | *Fagus sulvatica* | Switzerland | AJ302469 | NA |
| ***Xylaria hypoxylon*** | **CBS 122620** | NA | Sweden | AM993141 | NA |

^1^ Acronyms: ATCC: ATCC: American Type Culture Collecton, Virginia, USA; BAFC: Herbarium, Department of Biological Sciences, Faculty of Natural Sciences, Buenos Aires' University, Argentina; CBS: Westerdijk Fungal Biodiversity Institute (CBS-KNAW Fungal Biodiversity Centre), Utrecht, The Netherlands; CFCC: China Forestry Culture Collection Centre, Beijing, China; CMM: Culture Collection of Phytopathogenic Fungi “Prof. Maria Menezes”; CPC: Culture collection of Pedro Crous, The Netherlands; HUEFS: Herbarium of the State University of Feira de Santana; HVFIG: Hoosic Valley Family Interest Group; IRAN…C: Iranian Fungal Culture Collection, Iranian Research Institute of Plant Protection, Tehran, Iran; IPV: Instituto di Pathologia Vegetale, Milan, Italy, and E. and J. Gallo, Modesto, CA; MFLU: Mae Fah Luang University herbarium, Thailand; MFLUCC: Mae Fah Luang University Culture Collection, Thailand; NFCCI: National Fungal Culture Collection of India, India; STEU: Department of Plant Pathology, University of Stellenbosch, South Africa; UCD: University of California, Davis; UFMGCB: Culture Collection of Microorganisms and Cells of the Universidade Federal of Minas Gerais; NA: not applicable. All the new isolates used in this study are marked by an asterisk (*). Ex-type strains are in bold, type species are denoted with the superscript “TS” and the disputable type species are denoted with the superscript “TSQ”.
